# Transdermal Minimally Invasive Optical Multiplex Detection of Protein Biomarkers by Nanopillars Array-Embedded Microneedles

**DOI:** 10.1021/acsnano.4c11612

**Published:** 2024-10-28

**Authors:** Adva Raz, Hila Gubi, Adam Cohen, Fernando Patolsky

**Affiliations:** † Department of Materials Science and Engineering, The Iby and Aladar Fleischman Faculty of Engineering, Tel Aviv University, Tel Aviv 69978, Israel; ‡ School of Chemistry, Faculty of Exact Sciences, Tel Aviv University, Tel Aviv 69978, Israel

**Keywords:** biomarkers detection, transdermal, nanopillars array, minimally invasive, bloodless

## Abstract

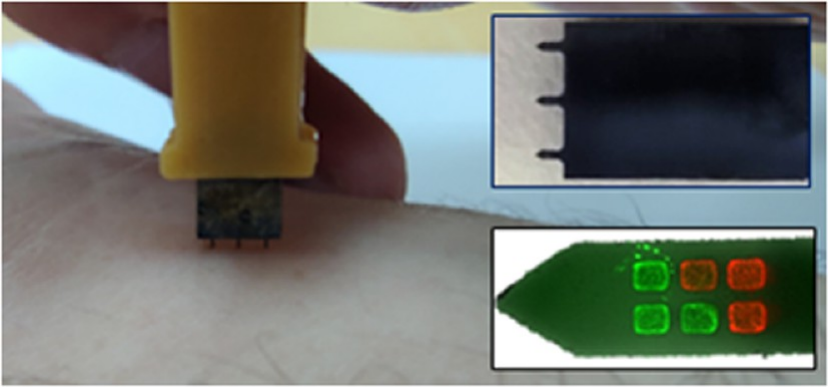

Biomarkers detection has become essential in medical diagnostics and early detection of life-threatening diseases. Modern-day medicine relies heavily on painful and invasive tests, with the extraction of large volumes of venous blood being the most common tool of biomarker detection. These tests are time-consuming, complex, expensive and require multiple sample manipulations and trained staff. The application of “intradermal” biosensors utilizing microneedles as minimally invasive sensing elements for capillary blood biomarkers detection has gained extensive interest in the past few years as a central point-of-care (POC) detection platform. Herein, we present a diagnosis paradigm based on vertically aligned nanopillar array-embedded microneedles sampling-and-detection elements for the direct optical detection and quantification of biomarkers in capillary blood. We present here a demonstration of the simple fabrication route for the creation of a multidetection-zone silicon nanopillar array, embedded in microneedle elements, followed by their area-selective chemical modification, toward the multiplex intradermal biomarkers detection. The utilization of the rapid and specific antibody–antigen binding, combined with the intrinsically large sensing area created by the nanopillar array, enables the simultaneous efficient ultrafast and highly sensitive intradermal capillary blood sampling and detection of protein biomarkers of clinical relevance, without requiring the extraction of blood samples for the *ex vivo* biomarkers analysis. Through preliminary *in vitro* and *in vivo* experiments, the direct intradermal in-skin blood extraction-free platform has demonstrated excellent sensitivity (low pM) and specificity for the accurate multiplex detection of protein biomarkers in capillary blood.

## Introduction

Biomarkers detection has become an essential tool in medical research and practice, aiding in the diagnosis of diseases, monitoring treatment effectiveness, and identifying potential drug targets. However, modern-day medical diagnostics still faces significant challenges, such as limited sensitivity and specificity, high cost, technical complexity, low sampling-to-detection cycle turnover, and so on, which can impede their efficacy and practicality in patient care.^1−4^ Despite the notable advances in medical diagnostics, there is an ongoing enormous need to address these challenges in order to enhance their applicability in the medical industry. Biomarkers, as quantifiable indicators of biological processes, hold significant potential in providing essential information about the health status of patients. By overcoming these challenges, biomarkers and other diagnostic tools can facilitate more accurate disease diagnosis and personalized treatment strategies.

Traditional laboratory-based procedures for protein biomarker identification can be time-consuming and invasive to acquire a sufficient amount of blood. Moreover, good medical care can be expensive, promote the risk of infections to the patient or sample hemolysis, and require specialized equipment and skilled personnel.^5,6^ These factors make it challenging to implement POC testing using the current methods of blood extraction, particularly in resource-limited or remote settings. POC testing tentatively provides an alternate technique, allowing for the detection of protein biomarkers faster, cheaper, and at the patient’s bedside or in the doctor’s office, even at home settings, without the need for laboratory equipment or specific training.^7,8^ Many POC diagnostics rely on the sampling of blood by venipuncture, which is a procedure that requires a healthcare professional and can be painful and uncomfortable for patients.^9−11^ The limits of the present technologies can be overcome by creating alternative blood extraction-free approaches, such as on-chip nanostructured devices. Furthermore, the simultaneous measurement of multiple bioanalytes in a single sample is highly desirable, offering fast, inexpensive, and reliable quantification. Therefore, applicable multiplexed POC testing, which tackles all of the aforementioned challenges, has become a major point of interest and serves as a holy grail for medical diagnostics.

To address these issues, capillary blood sampling has been proposed as a minimally invasive alternative to venipuncture for POC diagnostics. It can be extracted by individuals themselves, which can increase accessibility to testing and eliminate the need for healthcare professionals.^12−14^ However, capillary blood samples have been associated with varying biomarker concentrations, which may limit the accuracy and reliability of the POC diagnostic tests. Furthermore, numerous issues arise when minimal volumes of bodily fluids are extracted for diagnostic testing, namely, uncontrollable detrimental effects that can occur during extraction and postextraction manipulation steps, such as clotting and hemolysis.^15−17^ These effects can negatively impact the quality and accuracy of the sample, particularly before it reaches the final sensing phase. As a result, it is often not possible to perform multiplexed biomarker analyses on these small-volume samples, which can impede diagnosis and lead to significant analytical artifacts.

Analyzing capillary blood samples was recently shown to be possible via microneedle-based systems due to their direct and minimally invasive whole-blood extraction approach. As a minimally invasive technique, the microneedle insertion is almost painless and devoid of discomfort, ensuring a positive patient experience during the sampling process.^18^ Moreover, the tiny needle minimizes tissue trauma, thereby reducing the likelihood of complications such as hemolysis and the risk of infection throughout their use.^19−23^ The direct detection of capillary whole blood can eliminate the need for traditional laboratory equipment for the sample manipulation and pretreatments, thereby enhancing healthcare accessibility and facilitating point-of-care testing.

The latest developments include new apparatuses including multiplexed arrays using microelectronics,^24^ glucose level monitoring in diabetic individuals,^25−27^ biomarkers detection in interstitial fluid^28−30^ and more.^19^ Multiplexed microneedle arrays are engineered to collect multiple biomarkers simultaneously, reducing the number of samples required, while improving diagnostic accuracy, and making them useful for point-of-care testing. These technological developments have the potential to revolutionize healthcare by allowing for minimally invasive and rapid monitoring of biomarkers in whole capillary blood, leading to earlier disease detection and personalized treatment. Notably, most of the previously mentioned paradigms are microelectronic-based platforms which can measure changes in the electrical properties of the sensing surface upon biomolecules binding. The major advantage of electronic biosensors is their high sensitivity, rapid response, and good linearity, theoretically allowing for excellent protein detection outcomes.^20^ However, several disadvantages plague electronic biosensors, making them inadequate for universal applications. Namely, they can be susceptible to signal drift and interference, which can result in inaccurate readings and require frequent calibration. Additionally, electronic biosensors may require complex fabrication and processing procedures, leading to high costs and limited scalability for mass production.^31,32^

In this context, optical-fluorescence-based biosensors rely on a different approach, utilizing the fluorescence emission of a probe upon interaction with the target molecule. In contrast to electronic biosensors, they offer high sensitivity and selectivity, as well as stability and the ability to detect and quantify a wide range of targets. Fluorescence-based biosensors also have a wide dynamic range, enabling the detection of low-abundance targets in complex biological samples.^33,34^ Another advantage of fluorescence biosensors is their compatibility with microfabrication techniques, which allows for the development of miniaturized devices for point-of-care and field-based applications with a simple operation. Lately, new and improved techniques for on-site fluorescence microscopy are being developed including smartphone spectroscopy, enabling on-site cheap fluorescence detection without the use of external power.^35−37^ In contrast, electronic biosensors may be limited by their need for external power sources.^38^

In a prior study conducted by our group, the notable efficacy of vertical arrays of silicon nanopillars (SiNPs) for the rapid separation and sensing of target proteins from complex biosamples was demonstrated.^39−41^ The SiNPs platform was created via metal-assisted chemical etching (MACE), to create a stable nanostructured surface directly from a silicon wafer. MACE is a simple and low-cost method for fabricating Si nanostructures, with the ability to control their shape, diameter, length, and nanostructure orientation relative to the substrate.^42,43^ The resulting SiNPs arrays provide several advantageous features, such as a sensing area for biosensing applications and especially immunosensors. For example, a high surface area-to-volume ratio renders them highly sensitive to changes in the local environment, a crucial characteristic for detecting small amounts of analytes and enhancing the resulting analytical signal.^33,44−46^ Furthermore, the 3D architecture of SiNPs arrays creates organized cavities with limited protein diffusion. This results in a substantially lower diffusion mean-free path for the confined proteins, forcing them to linger inside the cavities as they are being repeatedly adsorbed to the surface-anchored antibodies on the pillar surfaces within the limited interpillars region. Hence, improving the accessibility of analytes to the sensing elements, amplifying their sensitivity.^39^

Herein, a fully integrated array-like device is presented, which combines the advantageous architectures of both microneedle-like devices and SiNPs ultraefficient biomarkers collecting elements as the sensing area. The simple fabrication process of the device results in a fully embedded SiNPs array on the surface of the needles, while each sensing sub-area of the array can be further chemically functionalized with specific antibodies, targeting specific proteins and biomarkers. The needle-embedded nanopillar array allows direct, rapid, and minimally invasive capillary whole-blood sampling, while the sensory area of the functionalized embedded SiNPs serves as an excellent bed for immediate analysis of the sample via fluorescence microscopy. The device’s diagnostic clinical capability for highly sensitive, selective multiplex detection of protein biomarkers, such as prostate-specific antigen (PSA), which was selected as a preliminary proof-of-concept candidate, was demonstrated in this study. These findings will allow the development of future minimally invasive applications for the detection of additional biomarkers related to various diseases of interest.

## Results and Discussion

**Figure 1:**
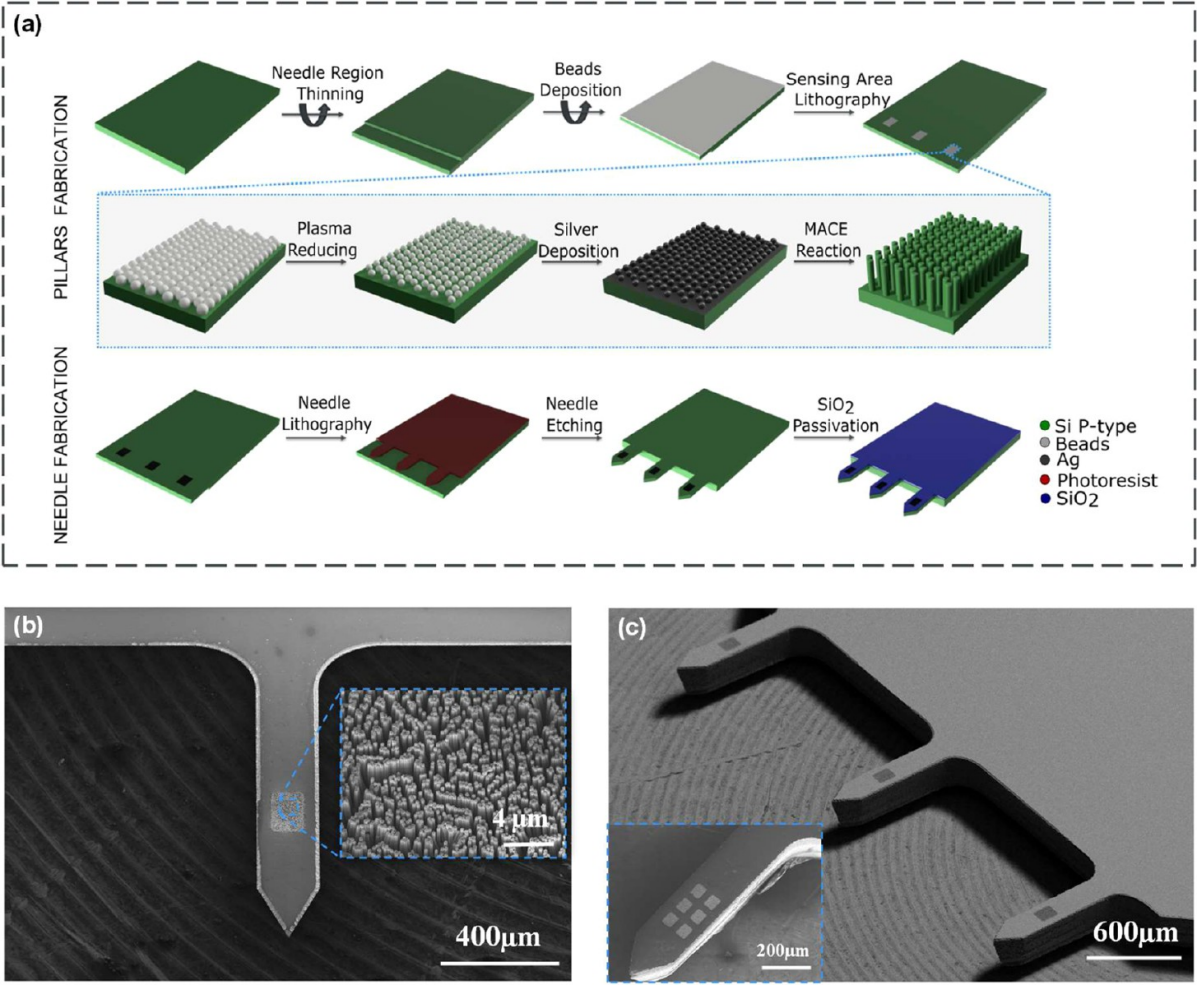
Fabrication and characterization of the SiNPs-based microneedle array. (a) Schematic illustration of the fabrication process. (b) SEM images of the fabricated needles with the SiNPs array; the dimensions of the needles are 220 μm in width and 1000 μm in length, while the sensing area is 120 by 150 μm, respectively. The blue inset shows a higher-magnification image of the vertically aligned and ordered SiNPs sensing array. (c) SEM images of the entire microneedle device presenting the three-needle platform, with each needle element containing one sensing area. The blue inset shows an alternative fabrication design including six sensing areas on a single needle structure, for the performance of multiplex detection on a single needle element.

A SiNPs array-embedded silicon microneedle device was created to support the monitoring of physiological parameters through the skin. Intradermal monitoring requires a robust sensing device that will stay mechanically and chemically stable during its implementation.^24,47,48^ The fabrication process of the microneedle platform is schematically depicted in Figure 1a. A P-type doped native silicon oxide wafer was selected with a crystallographic orientation of (100). The crystallographic orientation of the silicon wafer is of paramount importance in creating vertically aligned SiNPs using the anisotropic etching process of the MACE reaction.^42,43^ Prior to the fabrication process, and in order to prevent damage to the nanostructures, the backside of the needle region undergoes mechanical thinning using a dicing saw. The SiNPs were fabricated by the deposition of a monolayer of polystyrene beads, 500 nm in diameter, using a spin-coater on the silicon wafer, serving as an etching mask for the MACE reaction To reduce the diameter of the beads down to 300 nm, and create a beads array with an interdistance of 200 nm, the beads were subjected to oxygen plasma etching.^49^ Then, 45 nm of silver was deposited on top of the etched polystyrene beads as a catalyst for the wet etching reaction. The sensing area was protected and defined via UV lithography, allowing for the removal of the silver and beads from unprotected areas with wet etchers and oxygen plasma, respectively. The vertical SiNPs array is formed using the silver catalyst and a HF/H_2_O_2_ mixture as the etchant and oxidating agent, respectively. The final SiNPs array consists of pillars with heights ranging from 5 to 15 μm, Figure S1 depicts a focused ion beam (FIB) cross-section of the fabricated SiNPs after sputtering.

To acquire the final structure of the sharp tip microneedle for easy penetration of the skin, a deep reactive ion etching (DRIE) approach was used. The SiNPs structure, together with the thin edge of the needle, is prone to high-energy ion damage, which will result in structural defects. The needle region thinning, previously presented, reduces the etching time and prevents structural damage. Once the entire device is ready, a SiO_2_-protective layer was deposited using plasma-enhanced chemical vapor deposition (PECVD). The silica layer with a thickness of approximately 20 μm was fabricated as a 150 μm × 120 μm pool. Energy-dispersive X-ray spectroscopy (EDS) analysis of the compounds on the surface of the needle is presented in Figure S2, showing the silicon oxide window around the SiNPs sensing area. Moreover, a scanning electron microscopy (SEM) image using the backscattered electron detector (BSD) depicted in Figure S3 shows the shadowing effect surrounding the sensing window due to the added 20 μm of silica. This design was implemented to ensure accessibility to the sensing area while maintaining the integrity of the microneedle region. The silica pool is an important step in the design of a functional microneedle area, thus, the protective layer serves numerous functions, starting with addressing the potential contamination encountered during the penetration of the skin.^50^ Additionally, the insertion of the sensing elements into the intradermal layers can result in mechanical abrasion and subsequent detachment of the covalently attached molecular biorecognition layer.^21,50,51^ By etching the silicon wafer, the pillars are formed in a buried configuration, with their tips positioned at the surface level, while being shielded by the bulk of the wafer. Furthermore, the incorporation of several micrometers of silica provides supplementary safeguarding for the pillars mechanical integrity during the insertion process. Hence, the elevation of the surface height *via* the silica layer represents an indispensable and pivotal factor for the successful execution of blood extraction-free, intradermal protein detection. The final microneedle-embedded pillars are shown in the SEM image provided in Figure 1b, and the blue inset represents a higher magnification of the SiNPs sensing area. Within the scope of Figure 1a, a single sensing area is fabricated on each needle element, as depicted in Figure 1c. The maximum amount of sensing areas for each needle is solely limited by the dimensions of the needle itself, and the desired size of the individual sensing sub-areas. Therefore, the direct fabrication of multiplex devices becomes feasible, thereby enabling enhanced redundancy in the resulting sensing capabilities. Figure 1c illustrates the fabrication of six distinct sensing sub-areas in a single needle element, demonstrating the potential of our platform.

To receive the most effective sensing devices, the exact length of the microneedles should be determined. Previous work of the group, as well as additional research on that topic, showed that 1000–1500 μm microneedle is the optimal needle length range to reach and rupture intradermal blood capillaries networks, while maintaining minimal discomfort for patients.^24,52^

The monitoring and quantification of numerous protein biomarkers are critical for the early identification of a wide spectrum of diseases. Typically, this entails an invasive and unpleasant operation that involves the extraction of a few milliliters of venous blood for diagnostic purposes. Nonetheless, immense progress has recently allowed for a potential decrease in the necessity for this procedure. Rapid antibody–antigen binding followed by fluorometric intensity measurements can be applied with the use of small volume capillary blood samples. This method minimizes invasiveness and discomfort, while allowing for quick diagnostic analysis.

**Figure 2:**
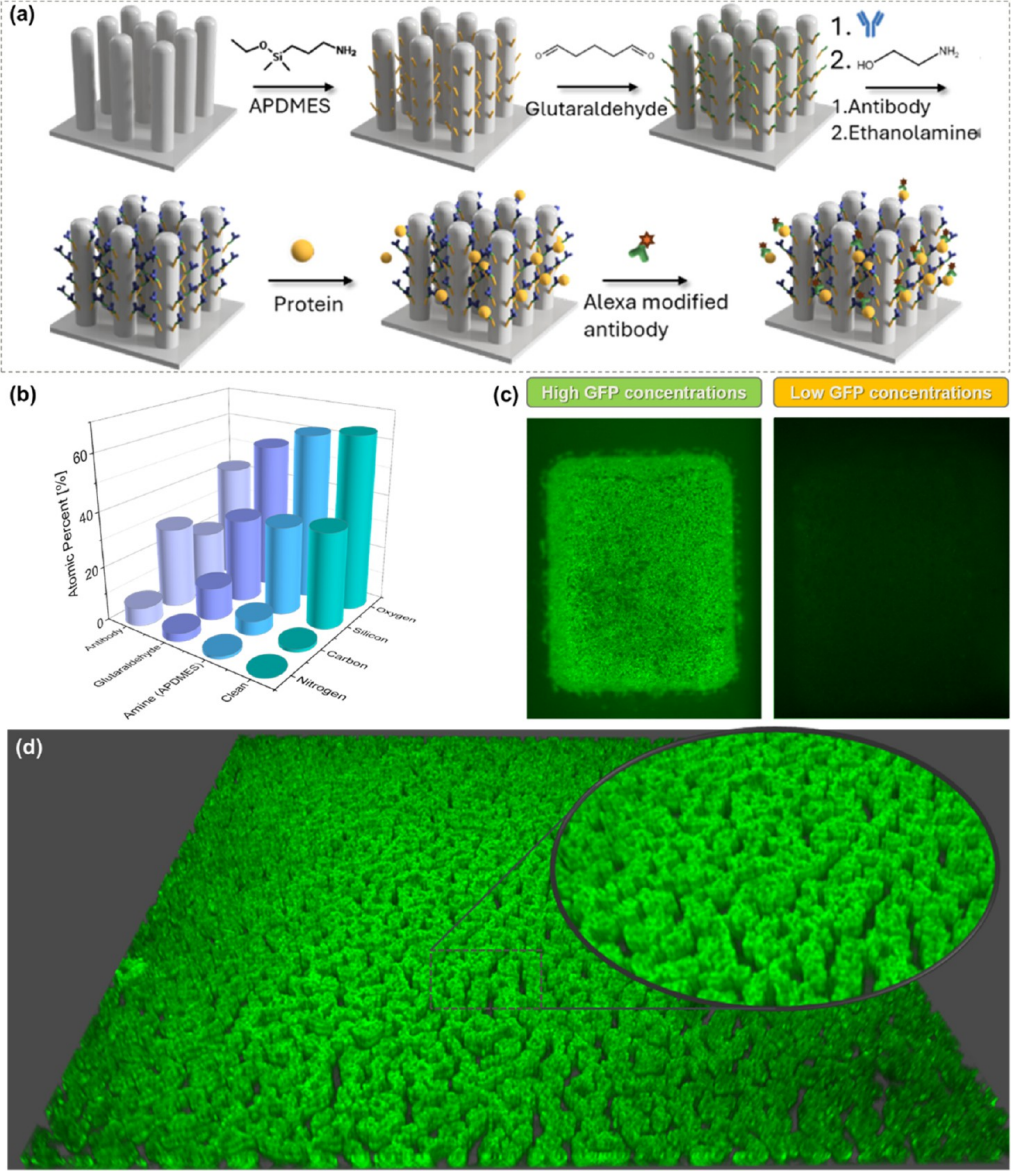
Surface modification process. (a) Schematic illustration of the chemical modification process and fluorescent labeling. (b) XPS results of the four different stages of surface modification. (c) Fluorescent microscopy images showing the concentration-dependent fluorescence intensities as a result of GFP protein binding to the anti-GFP modified surface. (d) Fluorescence imaging presenting GFP binding on the entire pillars’ height, 8 μm, by 3D imaging of the nanopillar sensing area (Z-stacking).

As proteins from the human body are mostly nonfluorescent, to first show the ability of the device, a modification of the anti-green fluorescence protein (GFP) antibody was conducted. In order to enable transdermal monitoring of different biomarkers and proteins, a chemical modification of the SiNPs surface is conducted as schematically illustrated in Figure 2a. First, the needles were dipped in a 3-aminopropyldimethylethoxysilane (APDMES) solution, an amino-silane derivative, for 2 h in an inert environment, followed by a thorough wash in toluene and IPA, and placed on a heating plate at 115 °C for 30 min for full dehydration to fully stabilize and enhance the covalent bond’s stability between the APDMES and the surface and evaporate all solvents.^53^ Subsequently, the needles were immersed in a solution consisting of 160 μL of a 10% glutaraldehyde solution in phosphate buffer containing 0.03 M of cyanoborohydride, acting as a reduction agent, for the duration of 1 h followed by a thorough wash in deionized water (DIW). The needles were then modified overnight with 40 μg/mL anti-GFP solution in phosphate buffer with 50 mg of cyanoborohydride at 4 °C. The yield of the binding between the antibody and the glutaraldehyde units is low, as some of the glutaraldehyde molecules on the surface remains unbound. To prevent nonspecific binding, blocking of unreacted aldehyde was performed by dipping the needles for 2 h in 160 μL of ethanolamine solution (400 μL in 25 mL phosphate buffer containing 50 mg of cyanoborohydride).^54^ An additional 2 h blocking step is performed by dipping the needle in 160 μL of skim milk in phosphate buffer silane (PBS) to minimize nonspecific adsorption to the biosensor surface that can interfere with detecting the target analytes.^55,56^ Consequently, the protein of interest is introduced to the chip in a buffer or Bovine serum-containing solution. For nonfluorescent proteins, the concluding steps in the detection process include submerging the chip in Alexa-modified secondary antibody, and evaluating the fluorescence signal with fluorescence microscope.

X-ray photoelectron spectroscopy (XPS) analysis was conducted for the different modification steps on a silicon wafer with the fabricated SiNPs, as presented in Figure 2b. For the clean silicon wafer, only silicon and oxygen elements from the native oxide layer are observed. Once the APDMES is covalently bonded, a rise in the carbon and nitrogen atomic concentrations is detected. Furthermore, a significant increase in carbon and nitrogen atomic concentrations occurs once the glutaraldehyde and IgG antibody molecules are bonded to the surface. The concentration of silicon and oxygen is reduced in every modification step, as the silicon substrate surface is covered and screened by the introduced organic compounds ad-layers. The XPS measurements show that the antibody modification process to the sensing device was successful. Full XPS spectra are shown in Figure S4.

The increase of the surface area owing to the nanopillars structure is fundamental to the sensing application. Figure S5 depicts a modified plain silicon wafer with the same process which yields poor sensing results, which are approximately in the device’s noise range and saturates very fast. This highlights the efficiency of the SiNPs in minimizing the reflected light. The nanostructures create a textured surface that significantly reduces reflectance; this increased absorption is attributed to the multiple scattering events within the nanostructures, which trap incoming light and reduce its escape. As a result, the noise level is lowered, enabling a much more sensitive analysis.

To further show the success of the modification process, the anti-GFP antibody chemically-modified microneedle array was incubated in the presence of the GFP protein. The first needle was dipped in a low concentration of GFP, 10 pM, while the second microneedle was incubated in a high concentration of GFP, 10 nM. Figure 2c presents fluorescence microscopy images showing the different contrasts in the images due to the difference in the fluorescence intensity. The microneedle introduced to high GFP concentration shows a high contrast, with a high-intensity fluorescence signal from the SiNPs active window, while the second microneedle shows low contrast between the SiNPs active window and its surrounding microneedle surface due to low fluorescence intensity. The different reactions demonstrate the successful antibody–antigen binding, and thus the successful antibody anchoring the SiNPS active surface, proving the successful chemical modification steps. Figure 2d shows the full modification of the pillars’ surface via 3D image using a fluorescence microscope and GFP protein binding (Z-stacking).

**Figure 3:**
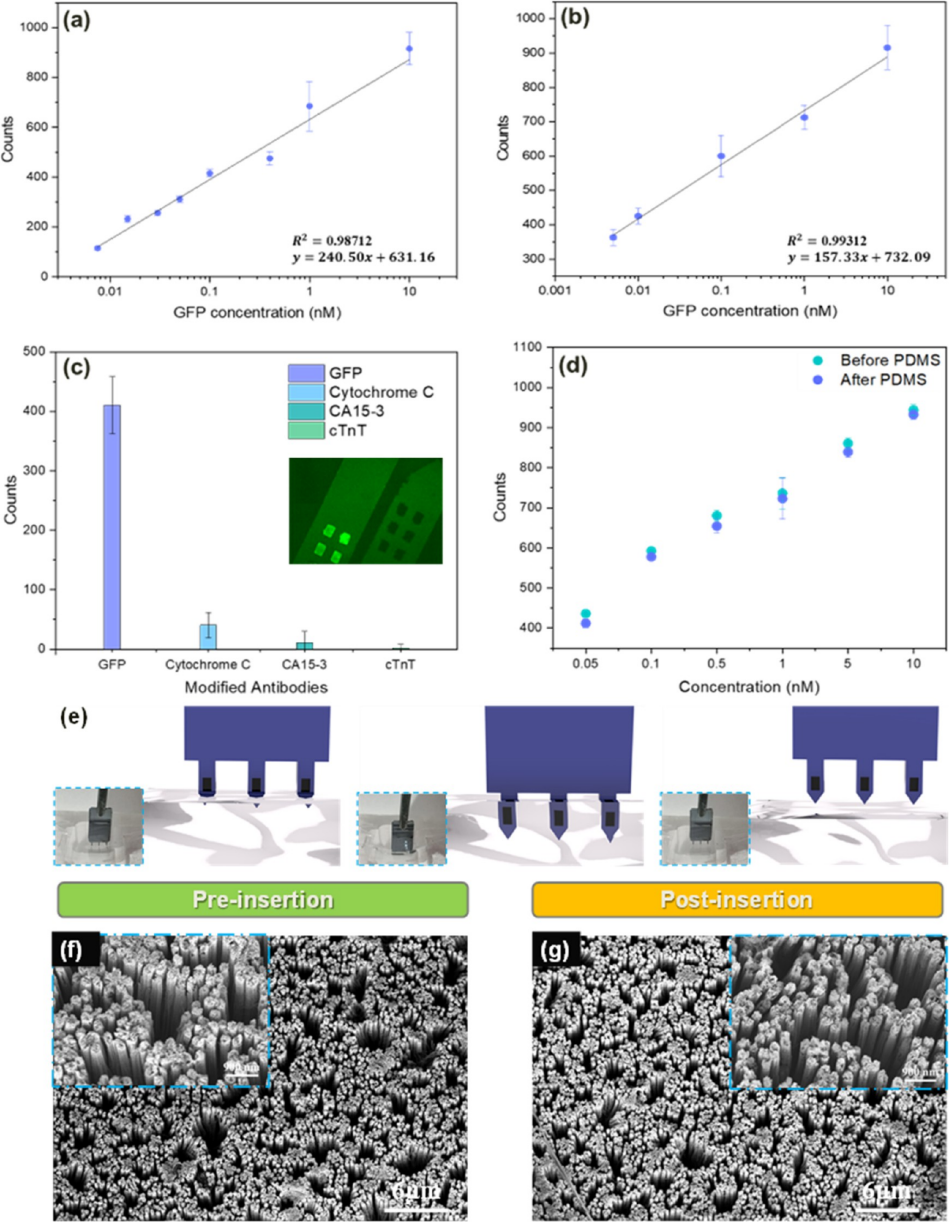
(a) Linear response curve with increasing GFP concentrations in spiked PBS buffer solutions. The intensity reading is the average of three areas (*N* = 3). (b) Linear response curve to increasing GFP concentrations in GFP-spiked bovine serum samples. The intensity reading is the average of three areas (*N* = 3). (c) Specificity measurements of four needles, each modified with different antibodies: anti-Cytochrome C, anti-CA-15-3, anti-cTnT, and anti-GFP, all incubated into the GFP protein sample. The results clearly show that only the needle modified with the specific anti-GFP antibody presents a high fluorescent response, while the nonspecific antibodies shows a negligible response. The inset shows an optical fluorescence image presenting the difference in fluorescence between the specific and nonspecific binding areas. (d) Fluorescence intensity measurements on the microneedle device sensing area, before and after PDMS insertion. The result shows similar intensity measurements between the two, proving no abrasion of the biorecognition layer due to the insertion occurs. (e) Schematic illustration of the “insertion and extraction” of the microneedle device from the solid PDMS sample. The blue insets show the respective optical images of the same process. (f) SEM image of the SiNPs sensing area before pricking of the PDMS. The blue inset shows a higher magnification of the area. (g) SEM image of the SiNPs after extraction from the PDMS showing no deformation of the nanopillars structure. The blue inset presents a higher magnification of the same area, proving that the nanopillars remain intact.

Preliminary fluorescence microscopy experiments were conducted to show a concentration-dependent behavior for the binding of the protein GFP, the samples were excited using a 470nm wavelength, and the fluorescence was measured at an emission range of 495–535 nm.^57^Figure 3a illustrates the linear response curves to different GFP concentrations in spiked PBS buffer samples. Similarly, Figure 3b shows measurements in bovine serum solutions spiked with increasing GFP concentrations. A clear linear increase in the fluorescence intensity was measured as a result of the specific binding of GFP to the surface of the antibody-modified SiNPs-embedded microneedle element. Signal normalization was performed against an unspiked solution. The response is taken as an average value of the entire sensing area. These results show a detection sensitivity and limit of detection (LOD) in the low pM range.

Specificity tests of the microneedle-embedded SiNPs array response were conducted by measurements in the presence of high concentrations of the nonspecific GFP biomarker, as shown in Figure 3c. The SiNPs array was modified with anti-Cytochrome C, anti-CA-15-3, and anticardiac troponin T (cTnT) antibodies, and showed a near to zero response to the introduced highly concentrated GFP protein. On the other hand, the anti-GFP-modified SiNPs array showed high fluorescence intensity in the presence of the GFP protein. These results indicate the high specificity of our sensing microneedle devices for the specific detection of the desired protein biomarker. This concept is presented in the fluorescence measurements in Figure 3c (inset). The two microneedles were introduced to the GFP protein solution, while the left needle modified with anti-GFP showed a response to the GFP protein, the right needle modified with anti-cTnT showed no response to the nonspecific GFP protein.

The safety and mechanical integrity of the SiNPs sensing area within the silica pool are of utmost importance for the final clinical application of the sensing platform, and were further explored by a series of additional tests. Figure 3d presents fluorescence measurements of the microneedles incubated with increasing GFP concentrations, before (cyan dots) and after “insertion-and-extraction” (purple dots) fom a solid poly(dimethylsiloxane) (PDMS) sample, a skin-mimicking slab pricking process. The graph shows that the fluorescence intensity values do not change as a result of the microneedle insertion into the PDMS block, demonstrating the absence of any mechanical abrasion on the covalently attached sensing layer during insertion and removal. Moreover, these tests were conducted to provide conclusive evidence regarding the robust protection of the SiNPs array, and to ensure their structural preservation during insertion and removal. Figure 3e presents optical images, together with a schematic illustration, of the insertion and extraction processes of the microneedle array. The full insertion process is featured in Movie S1, which demonstrates that all three microneedles on the device are fully intact following this process. Figure 3f,g depicts SEM images before and after the insertion, respectively, proving that there are no structural defects or broken pillars as a result of the insertion step. The insets of Figure 3f,g present higher-magnification SEM images of the SiNPs elements, showing that PDMS did not leave any residue on the array.

Aside from acting as a protection layer for the biorecognition layer, the silica deposited protective layer creates a 20 μm window, allowing fast and full wetting of the sensing area when introduced to the analyzed sample medium. By skin pricking, a capillary blood “pool” rapidly forms and collects inside the sensing area window.^24^

Figure S6 and Table S1 show the repeatability between three microneedles on a single chip, and the reproducibility from multiple chips, respectively. The plot presents the fluorescence from each needle in response to increasing concentrations of GFP. It is evident that all three microneedles from the same device, as well as needles from different chips, react similarly inside the error range. Therefore, the microneedles arrays are highly reliable and produce an extremely reproducible response.

Essential requirements for microneedles sensors that operate *in vivo* are their biocompatibility and mechanical stability. While the skin is pricked, the microneedles reach the dermis layer for analysis of the capillary blood. It is crucial to ensure that no silicon residues remain in the skin after the pricking process, as those could lead to inflammatory reaction or skin irritation.^58^ Previously conducted experiments in our group, with identical needle shape devices, concluded that the force required for skin insertion is between 0.2 and 1 N, while the designed microneedles can endure vertical force up to 5 N, thus ensuring the integrity of the needle during the skin sampling procedure.^25^

Following the mechanical tests, the silicon nanopillar’s endurance was assessed along with the device’s biocompatibility. A skin sample was pricked with a modified array, and fluorescence was measured before and after insertion to confirm the integrity of the surface bound molecular layers. Subsequently, SEM imaging was used to assess the integrity of the nanopillars. The results in Figure S7 exhibit the effect of the pricking procedure on a needle with the SiO_2_ layer. As shown there, the pricking procedure had no impact on the nanopillars, and no probe detachments occurred during skin insertion.

**Figure 4:**
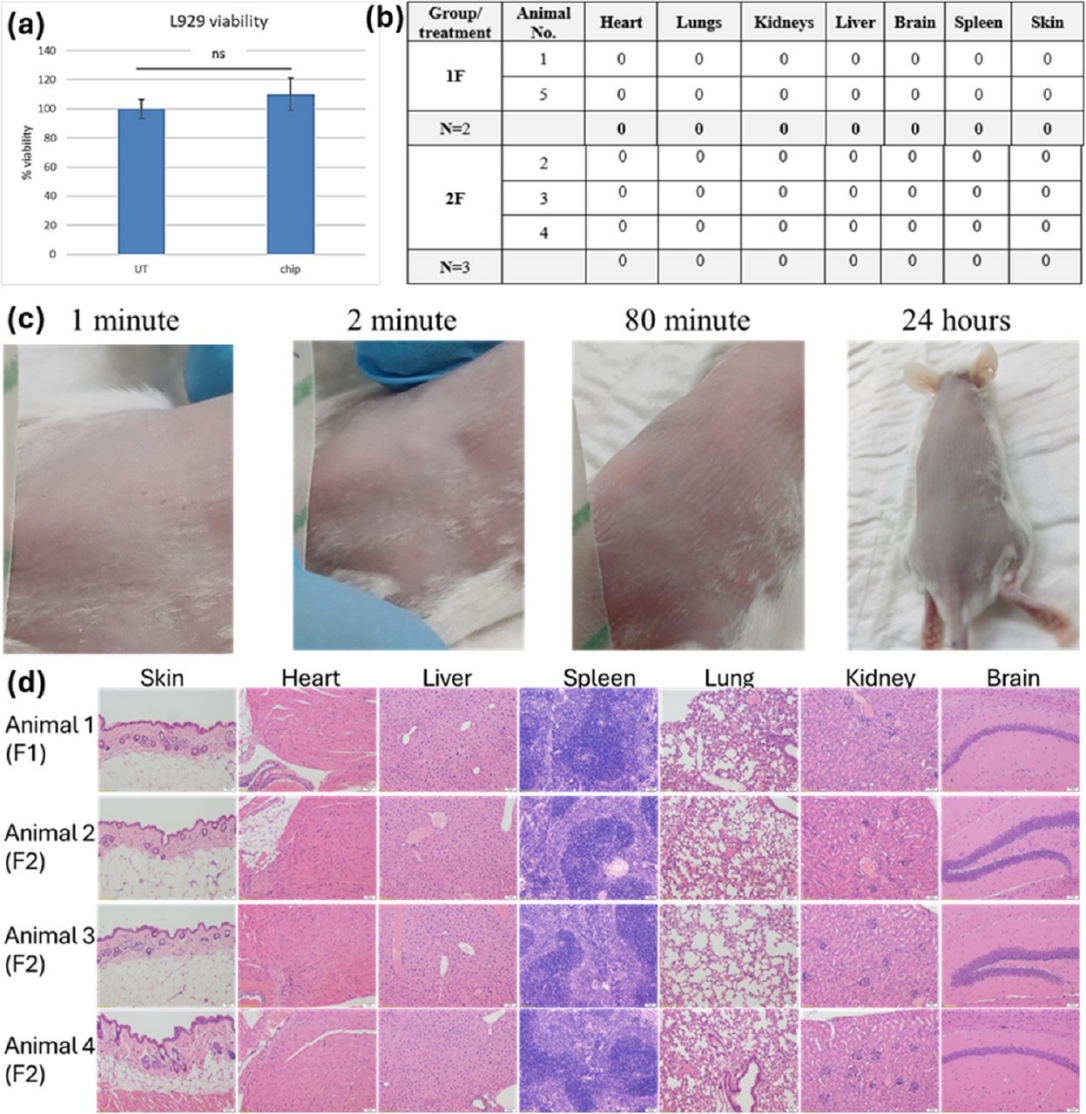
*In vivo* and *in vitro* biocompatibility results. (a) Effect of the microneedles on L929 cells viability. Left and right columns show the results of the untreated and treated cells, respectively. (b) Summary of the semiquantitative analysis of the histological findings, using a scoring method of five grades (0–4), for the severity of the pathological changes, when grade 4 indicates severe pathological findings. (c) Test subjects’ images directly after, 2 min, 80 min, and 24 h after pricking. (d) Histological photographs of H&E-stained organs.

Biocompatibility evaluations on the SiNPs-embedded microneedles were performed, both *in vivo* and *in vitro* to ensure the safety of the sensors for future applications. Mouse fibroblast cells (L929) were cultured for 24 h, with or without SiNPs-embedded microneedles. After culture medium exchange and sensors removal, a viability qualitative evaluation of the cells was performed by microscopic grading. Additionally, an XTT assay was performed for more precise inspection. As seen in Figure 4a, no significant differences were observed in the viability of untreated cells and cells incubated with the microneedles. In addition, microscopic evaluation resulted in 0 according to the grading system (Supporting Information), both for the control cells and for incubated cells.

To conduct a more thorough examination of the array’s biosafety, an *in vivo* experiment was conducted. Modified SiNPs-embedded microneedles were inserted into the back skin of 5–6-week-old, female, ICR mice for 1 min. The needles were either chemically-modified or non-chemically modified, in order to evaluate the bare nanopillars and the modified nanopillars’ safety independently. During the pricking procedures, all the needles remained intact. Photos of the pricked skin were taken right after pricking, following area wiping, 20 and 80 min after pricking and before termination, 24 h later. Two minutes after the procedure, the pricking holes on the subject’s skin were already barely visible, as seen in Figure 4c. Sections of the animal’s organs (heart, lungs, kidneys, liver, brain, spleen, and skin) were stained with H&E and evaluated for any histopathological findings. Microscopic evaluations of the stained organs and skin resulted in no physical or immune-inflammatory findings. In addition, Figure 4b,d presents that none of the study subjects exhibited adverse clinical signs during the study until termination, and no apparent lesions or any histopathological changes were observed on any of the examined organs after termination. The full clinical report is attached as Supporting Information.

Together these results show that the sensing microneedles have no effect on the viability of the cells and the health of mice. Furthermore, no immuno-inflammatory responses were detected in the pricked mice skin. The great compatibility of the silicon NPs-embedded microneedles indicates great potential for future clinical applications.

While the preliminary concept and fluorescence measurements were conducted using GFP, subsequent measurements were performed on real human serum samples for quantifying levels of the protein biomarker PSA in capillary blood. Prostate-specific antigen (PSA), a protein biomarker originating from the prostate gland and found in seminal fluid, has emerged as a pivotal component in the detection of prostate cancer. Elevated PSA levels in blood can serve as an indication of prostate cancer. Normal PSA levels in healthy men range up to 4 ng/mL, with values exceeding this threshold raising concerns about potential prostate cancer.^59,60^ Serum PSA levels in females are generally considered negligible; however, breast tissues can also produce PSA. Elevated PSA levels in females serum may be indicative of conditions such as breast cancer, cysts, or fibroadenomas.^61,62^ Consequently, the detection of PSA in blood has become an increasingly prominent in medicine, diagnostics, and scientific research.

Prior to *in vivo* intradermal measurements in capillary blood, the sensitivity of the microneedle device toward the protein biomarker PSA was evaluated through *in vitro* measurements.

**Figure 5:**
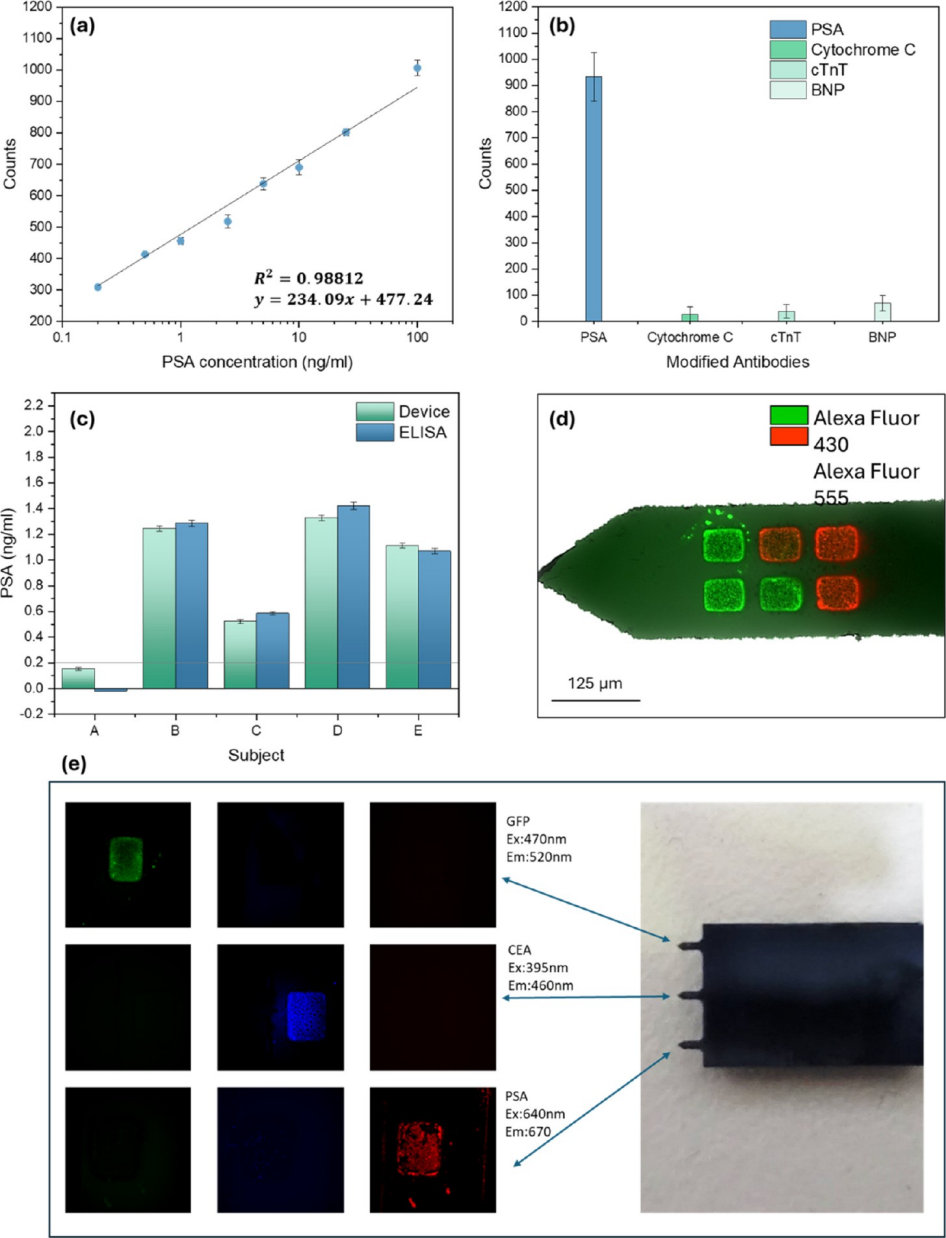
(a) Linear response curve to increasing PSA concentrations in spiked bovine serum samples (*N* = 10). (b) Specificity measurements (*N* = 10 measurement repetitions on a single device for 10 different devices), each modified with PSA antibody and introduced to different proteins solutions: Cytochrome C, BNP, cTnT, and PSA. The results clearly show that only the specific PSA protein biomarker leads to a high fluorescent response, while the nonspecific proteins show a negligible response. (c) *In vivo* measurements in five human volunteers for quantification of capillary blood PSA concentration using the SiNPs device (cyan bars, *N* = 3), in comparison to venous blood-based ELISA measurements (blue bars). (d) Fluorescence image of the multiplex detection of two different fluorophores on the multiple sensing area device. (e) Fluorescence image of multiplex detection with three different antigens on the same chip.

A concentration-dependent sensing behavior was observed; Figure 5a demonstrates a linear response to different PSA concentrations in spiked untreated bovine serum samples, showing sub ng/mL (low pM) sensitivity, with a LOD of 0.2 ng/mL. Additional specificity tests of the SiNPs platform were performed using high concentrations of the nonspecific proteins cytochrome C, cTnT, and BNP, as shown in Figure 5b. The concentrations were 100 ng/mL for all tested proteins, including PSA, which is an order of magnitude higher than the physiological PSA level in a healthy human. The anti-PSA-modified array showed near-zero response to the highly concentrated cytochrome C, cTnT, and BNP, indicating the high specificity of the present sensing microneedle device for the specific detection of the PSA target biomarker. Furthermore, these results demonstrated low sensitivity to varying interferents (proteins, nucleic acids, lipids, etc.), as the measurements were conducted in untreated serum.

Following calibration of the sensing device *via in vitro* measurements, *in vivo* intradermal sensing measurements were performed on five human volunteers, by inserting the microneedle elements into the intradermal space for a short period of only 1 min. Figure 5c depicts the PSA concentration measurements of the tested subjects extrapolated from the device calibration presented in Figure 5a. The tested subjects were: Subject A is a healthy 25-year-old female, Subject B is a healthy 30-year-old male, Subject C is a healthy 25-year-old male, Subject D is a healthy 28-year-old male, and Subject E is a healthy 30-year-old male. The results show that the subjects’ PSA levels are within the normal healthy range for all male subjects. Very low PSA levels were measured for the female Subject A, lower than the lowest limit of the detection of the device, shown as the horizontal line, since normal PSA levels in healthy females are about 0.002 ng/mL.^63^ The accuracy of the PSA levels recorded from the *in vivo* microneedle measurements was further validated by performing gold-standard PSA-specific enzyme-linked immunosorbent assay (ELISA) tests, using venous blood samples from the same volunteers. The ELISA results depicted in the blue column in Figure 5c confirmed that the microneedles array *in vivo* experiments accurately measured the levels of the target PSA biomarker in blood, which directly correlates to the ELISA measurements. In addition, these experiments prove the excellent physiological correlation of PSA concentration levels between venous blood and capillary blood samples.

Nowadays new platforms of microneedle devices for POC applications offer the capability for multiplex detection of multiple protein biomarkers in a single test. On this device, each individual needle or sensing area can be modified with a different antibody according to specific requirements. Thus, this allows for fast and efficient multiplex detection. A highly accurate microdropper device (M2 Automation) was employed to label each needle on the same chip with a different antibody, to ensure the selectivity and visibility of the secondary antibodies when introduced simultaneously in a single cocktail. Here, CEA, PSA, and GFP antibodies were used during modification, and each secondary antibody has a different excitation and emission spectrum. The additional protein introduced here, CEA, shows the method’s reliability with different antibody pairs. The modified chip was submerged in a serum sample spiked with all of the respective proteins. After a simple wash with FPBS buffer, the chip was submerged in a secondary antibodies cocktail. Figure 5e presents the results obtained when analyzing the respective chip. These results ensure the method’s selectivity when introduced to different secondary antibodies simultaneously, and the ability to detect a single protein out of many.

Upscaling of the multiplex detection is also possible when labeling each sensing sub-area on the same microneedle as shown here with distinct fluorescent dyes. Specifically, half of the sensing sub-areas were labeled with Alexa Fluor 430, while the other half were labeled with Alexa Fluor 555. Utilizing a fluorescent microscope, the device was initially excited with the corresponding wavelength, implicit in its name, to excite the fluorophores, and their emission was observed using 495–535 and 575–615 nm filters, respectively. Figure S8 presents the raw images received during the acquisition. Figure 5d presents the results of the multiplex experiment, showing the specific fluorescent emission in each sensing area in response to the appropriate excitation.

**Figure 6:**
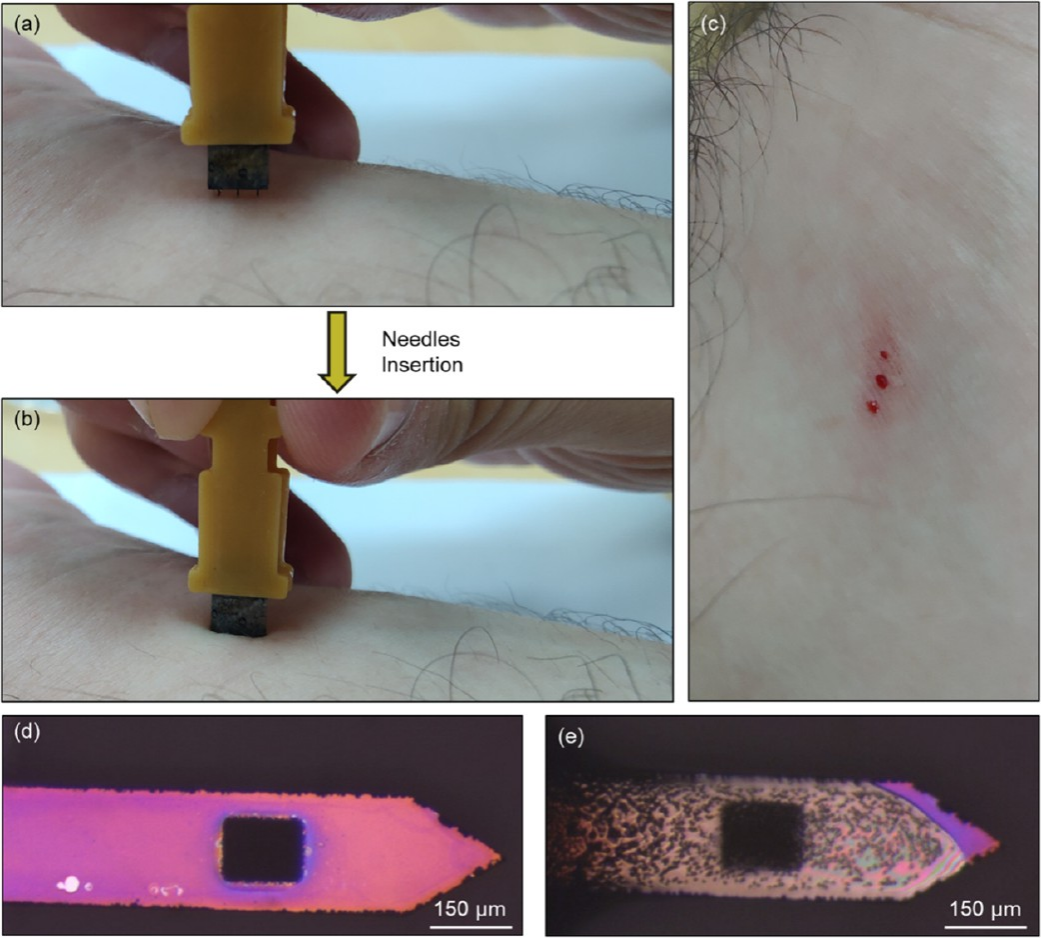
(a) Initial pricking step of the microneedle device connected to a 3D printed holder. (b) Fully inserted microneedle device inside a volunteer arm’s skin. (c) Three puncture holes for three needles on the device, with visible blood droplets as a result of the capillary network rupture during pricking. (d) Optical microscope image of the microneedle with the silica protective window before contact with the blood droplet. (e) Optical microscope image of the microneedle after contact with the blood droplet, showing red blood cells on the needle surface.

For the *in vivo* measurements, the device was connected to a handle support printed in a 3D printer to facilitate its precise, easy, and stable insertion. Figure 6a,b visually depicts the skin pricking process before and during insertion, respectively. Each microneedle on the device serves a crucial function in rupturing the capillary network creating individual blood pools. These blood pools subsequently fill the meticulously designed sensing area within the silica-protected window. Figure 6c presents the distinct blood pools generated by each microneedle as a consequence of the skin pricking procedure; each pool corresponds to a specific microneedle on the device chip. Figure 6d shows optical microscope images of the microneedle with the silica-protected layer before it contacts a blood droplet. The microneedle was then introduced to a blood droplet, evident by the red blood cells on the microneedle area, presented in Figure 6e. The device’s ability to accurately sense bioanalytes directly from capillary blood depends on the interface between the sample and the SiNPs array sensing area on the device. Movie S2 depicts the blood droplet draining into the depressed SiNPs array, fully wetting and saturating the sensing interface.

To further strengthen our claim, and to assess the discomfort endured during the pricking procedure with our microneedles, volunteers were pricked with five different types of needles and asked to rate the pain for each method on a scale from 1 to 10.

The pricking types were as follows: microneedle, 30G needle, 27G needle, 25 needle, and 23G. Each needle was inserted up to 1 mm inside different fingers to ensure identical conditions and prevent pain accumulation. The results depicted in Figure S9 prove that minimum pain was experienced when pricked with our microneedle elements. Moreover, volunteers reported lingering pain for 25G and 23G needles, and instant comfort when our microneedles were taken out of the finger.

To assess the chip applicability for fast POC testing, Figure S10 displays an additional experiment showing the signal saturation of GFP protein after less than 10 min, showing the possibility for a much shorter total analysis duration.

The blood extraction-free microneedle sensing platform presented in this study suggests a groundbreaking advancement in medical diagnosis, especially in POC testing. The current traditional laboratory-based medical diagnostics rely heavily on invasive, time-consuming, and expensive procedures involving extensive blood extraction and manipulation performed by trained professionals. Compared to traditional syringe-based *in vitro* intradermal test that usually takes a minimum of few hours and up to 3 days, our microneedles platform can perform the process in less than 20 min, with a better sensitivity and LOD, a few pM compared to 100 pM in traditional diagnosis.^64^ The low LOD is especially important for females, as their PSA normal level is extremelly low and any increase could indicate higher risk for breast cancer.^63,65^

The proposed diagnostic platform presented here utilizes chemically modified SiNPs-embedded microneedles, enabling intradermal penetration and quantitative sampling and detection of the desired biomarkers directly from capillary blood. This innovative POC paradigm offers minimally invasive, manipulation-free whole blood detection while exhibiting high sensitivity, specificity, fast detection turnover, and multiplexing capabilities.

## Conclusions

In conclusion, a microneedle-embedded SiNPs sensing array for the intradermal, minimally invasive, and blood extraction-free platform utilizing optical fluorescence measurements for the clinical POC detection of protein biomarkers is here demonstrated. By a fast, simple and cost-effective fabrication process, an array of vertically aligned SiNPs sensing areas is achieved by multiplying the surface area by 10-fold, together with the interpillar biomarker concentration phenomenon, resulting in a highly sensitive intradermal capillary blood sensing device. For the protection of the biorecognition layer, a silica layer surrounding the sensing area was implemented. This silica-protecting window allows no abrasion of the bonded antibody recognition layer from the sensing area, while it smoothly penetrates the skin to the desired depth. Additional durability tests show that no structural damage to the SiNPs array, or the microneedle itself, is observed as a result of the skin pricking process. The microneedle SiNPs array shows high specificity to the desired specific biomarkers, with a detection sensitivity limit in the low pM range. Moreover, fluorescence microscopy experiments showcase a clear and linear concentration-dependent sensing behavior to the target analytes, under the presence of highly abundant nonspecific protein potential interferents. Preliminary clinical tests were performed by the intradermal direct *in vivo* blood extraction-free detection of PSA on human volunteers *via* skin pricking. Our microneedle-embedded SiNPs sensing platform detection measurements correlate well with the values measured using venous blood from the same volunteers, through the use of gold-standard ELISA analysis. Moreover, additional multiplex chemical modification of two different fluorophores on a single microneedle proves the high POC testing abilities of our developed diagnostic method. Future integration of the platform with a portable optical readout system can further enhance the usability of this device, enabling various on-site and real-time clinical diagnostics. By utilizing minimal quantities of in-skin capillary blood, and the intrinsically rapid antibody–antigen binding reactions, coupled with fluorometric intensity measurements, this blood extraction-free microneedle sensing platform holds great promise for improving healthcare outcomes and patient access to advanced diagnostic tools.

## Materials and Methods

### Materials and Chemicals

In this study, the following materials and chemicals were obtained: polished Si wafer, P-type, (100), 380 μm (Silicon Valley Microelectronics), acetone (9005-68, J.T. Baker), isopropanol (9079-05, J.T. Baker), ethanol 97% (Bio-Lab), N-methyl-2-pyrrolidone (NMP, J.T. Baker), hydrogen peroxide (30% in water, Bio-Lab), methanol (HPLC, Bio-Lab), Triton X-100 (Sigma-Aldrich), sulfuric acid (95–98%, Bio-Lab), buffered oxide etchant 6:1 (BOE, Transene), hydrofluoric acid (48%, Sigma-Aldrich), hydrochloric acid (32%, Bio-Lab), gold etchant TFE (Transene), microparticles based on polystyrene (500 nm, 10% in DIW, Sigma-Aldrich), AZ-1518 (MicroChemicals), AZ-1505 (MicroChemicals), AZ-726 (MicroChemicals), PR1-12000A1 (FUTURREX), RD6 developer (FUTURREX), heatsink grease (Dow Corning 340 Heat Sink Compound Grease), deionized water (18 MΩ·cm), phosphate buffer (PB, 10 mM, pH 8.5), phosphate buffer saline (PBS, 10 mM, pH 7.4, with 2.7 mM KCl and 137 mM NaCl, Sigma-Aldrich), glutaraldehyde solution (50 wt % in H2O, G7651, Sigma-Aldrich), sodium cyanoborohydride (Angene), ethanolamine (98%, Sigma-Aldrich), (3-aminopropyl)-dimethyl-ethoxysilane (APDMES, 18306-79-1, Angene), anticardiac troponin T antibody (F24T19, HyTest), GFP protein (ab84191, ABCAM), anti-GFP antibody (ab1218, ABCAM), Zeba spin desalting columns (Thermo Scientific), anti-CA15-3 antibody (α Diognistic), anti-cytochrome C antibody (ab76237, ABCAM), dry skim milk powder (LAB-M), PDMS (Sylgard), PSA antibody pair (ab256313, ABCAM), PSA protein (ab78528, ABCAM), BNP protein (ab87200, ABCAM), cardiac troponin T protein (ab209813, ABCAM), Human Cytochrome C (ab131847. ABCAM), Alexa Fluor 647 (A-20186, Thermo Fisher), Alexa Fluor 430 (A-10169, Thermo Fisher), Alexa Fluor 555 (A-37571, Thermo Fisher), and Human PSA ELISA Kit (ab264615, ABCAM).

### Preparations of the Silicon Wafer

To prepare the silicon wafer for the needle and pillar fabrication, AZ-1505 resist was dispensed and spin-coated (500 rpm for 5 s and 4000 rpm for 45s) on the wafer for protection during the dicing process. A 3 in. p-type wafer was diced into 30 × 30 mm^2^ pieces using an automatic dicing saw (Disco DAD 3350). For the thinning of the needle area, AZ-1505 was spin-coated again on the front side of the silicon die, as the dicing was done on the backside. The dies were thinned on the intended needle areas to approximately 250 μm by lowering the saw up to the desired depth.

### Nanopillars Array Fabrication

First, a premade 400:1 methanol-Triton X solution was added to the polystyrene bead solution to create 1.3% polystyrene bead suspension, followed by the addition of a 5% volume of ethanol (97%) to the mixture. The suspension was dispersed by shaking for 10 min using a vortex. The 30 × 30 mm^2^ dyes were thoroughly cleaned with acetone to remove all resist residues, followed by immersing in fresh piranha solution (H_2_O_2_ 30%:H_2_SO_4_, 1:3) for 5 min, and a thorough washing with DIW and drying using an N_2_ gun. The surface was additionally cleaned and oxidized using an O2 plasma generator (100 W, 10 min).

The polystyrene beads suspension was spread evenly by dispensing 90 μL of the solution on the cleaned substrate and spin-coated (100 rpm for 60 s, 280 rpm for 35 s, 700 rpm for 40 s, 1200 rpm for 15 s). The polystyrene beads size was reduced to 300 nm diameter using PECVD plasma etching (50 sccm O_2_, 40 mTorr, 30 W, 7 min). This treatment is carried out to define the final radius of the pillars.^66^ A 45 nm silver film was thermally deposited onto the surface (0.2 Å/s).

For the fabrication of the desired sensing areas on the device, the silver and beads were removed using lithography techniques, leaving only the necessary sensor regions covered and protected. AZ-1518 photoresist was spin-coated and baked with the same parameters as before and exposed to a UV light with a dose of 45 mJ/cm^2^. The substrate was developed in AZ-726 developer for 1 min, and silver and beads were removed from the unwanted areas using gold etchant (TFE) for 30 s and O_2_ plasma etching (50 sccm O_2_, 40 mTorr, 30 W, 15 min), respectively.

The SiNPs formation was accomplished by wet etching the silicon substrate in an 8 mL solution of 7.6 M HF and 0.29 M H_2_O_2_ in DIW for 15 min followed by a thorough wash in DIW. Residues of silver and polystyrene beads were removed with HNO_3_ and O_2_ plasma, respectively (50 sccm O_2_, 40 mTorr, 30 W, 15 min). It is important to emphasize that an extended etching duration invariably leads to the formation of elongated pillars, which are characterized by an increased propensity to undergo warping and folding throughout the etching process. Consequently, such structural changes lead to the production of sensors with compromised functionality, thus rendering them ineffective for their intended purpose.

After the needle formation in the DRIE, a protective passivation layer of SiO_2_ was deposited on the microneedle device, leaving the sensing area clean. In order to protect the sensing area from the passivation, the AZ-1518 photoresist was spin-coated and baked with the same parameters as before and exposed to a UV light with a dose of 45 mJ/cm^2^. The substrate was developed in AZ-726 developer for 1 min, and the microneedle was placed inside the PECVD for the deposition (140 sccm N_2_O, 40 sccm 2% SiH_4_/Ar, 80 °C, 95 mTorr, 30 W bias 200W ICP, 1 h)

### Microneedles Fabrication

To protect the fabricated SiNPs from possible damage, a thick resist was applied before the deep reactive ion etching procedure (DRIE, Deep RIE Versaline DSE). PR1-12000A1 resist was applied and spin-coated at 300 rpm for 10 s and 3000 rpm for 40 s, then baked at 120 °C for 3 min. The etch mask was exposed to 480 mJ/cm^2^ divided into 5 short and consecutive exposures. Following exposure, the die was placed in RD6 developer for 8 min under constant rotation. BOE solution was used to remove the oxide layer from the unprotected silicon area. The substrate was then placed in DRIE and etched for 150 loops. The die was separated into individual devices using a dicing saw and placed in warm NMP to remove the remaining resist.

### Antibody Modification

Before the modification process, the microneedle device was placed in the PECVD for 15 min (200 sccm O_2_, 260 mTorr, 100 W) to remove all carbon content from the surface and to generate silanol groups on the SiNPs. In a glovebox under an Ar atmosphere, the device was placed in 200 μL of 95% APDMES solution for 2 h. The device was then submerged in 160 μL of toluene to remove any remaining APDMES solution, extensively washed with IPA, and heated at 115 °C for 30 min to completely evaporate any remaining solvents and to fully stabilize and enhance the covalent bonds between the APDMES and the surface.

Phosphate buffer (PB) was prepared by mixing 10 mM potassium phosphate monobasic solutions and 10 mM potassium phosphate dibasic solutions to pH 8.5. To bind the second linker, glutaraldehyde, 5 mL of filtered PB (FPB) with 50 mg of sodium cyanoborohydride was mixed with 1 mL of a 50% glutaraldehyde solution. The device was dipped in 160 μL of the prepared solution for 1 h and was consecutively rinsed with DIW.

The selected antibody was centrifuged in a desalting column to clean and purify it properly and was consequently diluted to 40 μg/mL for the modification using a prepared solution of 5 mL of FPB containing 50 mg of sodium cyanoborohydride. The microneedle array was dipped in 160 μL of the antibody solution and placed at 4 °C overnight on the SiNPs device.

A blocking solution was prepared by adding 100 mM ethanolamine to FPB with 50 mg of sodium cyanoborohydride, followed by a titration to maintain pH 8.5 using 32% HCl 32%. The unreacted aldehyde groups on the SiNPs were then blocked for 2 h using 160 μL of the mentioned solution. The sensor was then thoroughly washed by placing it in a 160 μL solution of clean FPB for 15 min. The final antibody modification step was the submergence of the device in a skim milk solution to eliminate all unspecified binding sites. This method was used to modify all of the antibodies used in this study.

### *In Vitro* and *In Vivo* Fluorescence Measurements

The *in vitro* measurements took place either in filtered phosphate-buffered saline (FPBS) or in bovine serum. The microneedle device was placed inside an Eppendorf containing 160 μL of either unspiked (“clean”) or protein-spiked solutions using different concentrations for approximately 1 h. The measurements were conducted using a fluorescence microscope with a 470nm LED and a 495–535 nm filter (LEICA MD4000 M fluorescence microscope with LEICA DFC450 camera).

PSA *in vitro* and *in vivo* measurements were done using Alexa Flour dyes. Following the capture antibody modification, the device was placed in 160 μL of bovine serum solution with different PSA protein concentrations. Consequently, the device was bound to 160 μL of PSA detector antibody previously labeled with Alexa Fluor 647 for 1 h and thoroughly washed in FPBS to remove the access antibody. The fluorescence intensity was measured using the fluorescence microscope with a 640 nm LED and a 495–535 nm filter.

*In vivo* measurements in capillary blood were performed similarly to the *in vitro* measurements replacing the spiked protein solution with a full penetration of the microneedle array into the volunteer’s skin with a 1 min wait inside the skin before removal.

### Antibody Alexa Labeling

PSA antibody pair was used for the PSA detection (ab256313). The detector antibody was labeled by using an Alexa Flour 647 labeling kit (A-20186). 100 μL of the detector antibody is added to the given Alexa and incubated for 2 h at room temperature with a gentle invention of the vial every 15 min to fully dissolve the dye. The purification steps involve the removal of unbound Alexa’s to the antibody. A spin column was placed in a 15 mL tube, and after stirring the purification resin, 1.5 mL of the suspension was added into the column and allowed to settle by gravity. The spin column was placed in the provided collection tubes and centrifuged for 3 min at 4000 rpm. 100 μL of the antibody bound to the Alexa was added to the center of the spin column, allowing the solution to absorb into the resin bed. The spin column was placed in an empty collection tube and centrifuged for 5 min at 4000 rpm. A small amount of the purified conjugate was diluted using 2 mL of FPBS.

The sensor was modified with the capture antibody as described above. After the device was introduced with the PSA protein, the detector antibody labeled with Alexa was incubated at 160 μL for 1 h.

### ELISA Measurements

ELISA kit to quantify total PSA was purchased from ABCAM (ab264615). The measurement protocol is as follows:

A 96-well plate coated with an antibody specific to Human PSA was used. A standard PSA solution of 80,000 pg/mL was diluted in Sample Diluent NS to perform calibration curve measurements of 62.5–4000 pg/mL as shown in Figure S11. The antibody cocktail was prepared using 300 μL of 10× Capture Antibody and 300 μL of 10× Detector Antibody with 2.4 mL of Antibody Diluent 4BI and mixing thoroughly and gently. 50 μL of standard solutions and samples were pipetted into the wells together with 50 μL of the antibody cocktail incubating for 1 h at room temperature on a plate shaker. The wells were washed thoroughly using 10% wash buffer PT, and then 100 μL of TMP development solution was added to each well for 15 min in the dark shaking, developing a blue color in proportion to the amount of PSA bound. 100 μL of Stop Solution changes the color from blue to yellow, and the intensity of the color is measured at 450 nm.

Venous blood was extracted and centrifuged to coagulate and remove the red blood cells. The test was performed directly on the separated plasma fluid remaining after diluting by a 4-fold in Sample Diluent NS provided in the kit.

### Material Characterization

Microscopy and EDS images were taken by using HR-SEM (Gemini 300, Zeiss). XPS measurements were carried out using a scanning 5600 AES/XPS multitechnique system (PHI). The SiNPs cross-sectional images were taken by ion sputtering the sample using a Thermo Fisher Helios 5 UC focused ion beam system (FIB).

## Supporting Information Available

FIB cross-sectional image of the SiNP using ion spattering; EDS characterization of the silica protection layer of nanopillars sensing area; BSD image of the needle showing shadowing effect around the SiNP sensing area as a result of the added protective layer of silica; full XPS spectra of the antibody modification process to the SiNP; concentration-dependent fluorescence measurement on a plain, GFP-modified silicon wafer; repeatability intensity measurements of the needles on a single chip under rising GFP concentrations; SEM imaging of the needles and the pillars post skin pricking; raw fluorescent images for multiplex detection; pain test survey presenting normalized pain reported from volunteers after finger pricking with the presented microneedle, 30G needle, 27G needle, 25 needle, and 23G; incubation time-dependence of binding of a secondary antibody to the protein on the pillars by fluorescence measurements; enzyme-linked immunosorbent assay calibration curve for PSA concentrations; fluorescence reproducibility from multiple chips with single GFP concentration (GFP concentration: 0.03 nM); and insertion process of the microneedle device in PDMS demonstrating all three needles on the device are fully intact following this process (PDF)
Blood droplet introducing to a needle (Movie S1) (MP4)
Microneedles device insertion and extraction from PDMS block (Movie S2) (MP4)
*In vitro* and *in vivo* evaluation of cytotoxicity and biocompatibility of microneedle devices (PDF)

## Notes

The research presented here complies with all relevant ethical regulations of the Tel Aviv University Ethics Committee. The human study protocol IRB 71.19.
