# Supplementary material for: Transdermal Minimally Invasive Optical Multiplex Detection of Protein Biomarkers by Nanopillars Array-Embedded Microneedles
Source: ACS Nano. 2024 Oct 28;18(44):30848–62. doi: 10.1021/acsnano.4c11612 (PMC11544710; doi:10.1021/acsnano.4c11612)
Supplement: Supplementary file 1 — nn4c11612_si_001.pdf [file nn4c11612_si_001.pdf]

Supplementary Section

Transdermal Minimally-Invasive Optical Multiplex Detection of Protein Biomarkers by Nanopillars Array-Embedded Microneedles

Adva Raz[1#], Hila Gubi[2#], Adam Cohen[1] and Fernando Patolsky[1,2*]

1. Department of Materials Science and Engineering, the Iby and Aladar Fleischman Faculty of Engineering, Tel Aviv University, Tel Aviv 69978, Israel.

2. School of Chemistry, Faculty of Exact Sciences, Tel Aviv University, Tel Aviv, 69978, Israel.

# Contributed equally.

Emails: fernando@post.tau.ac.il

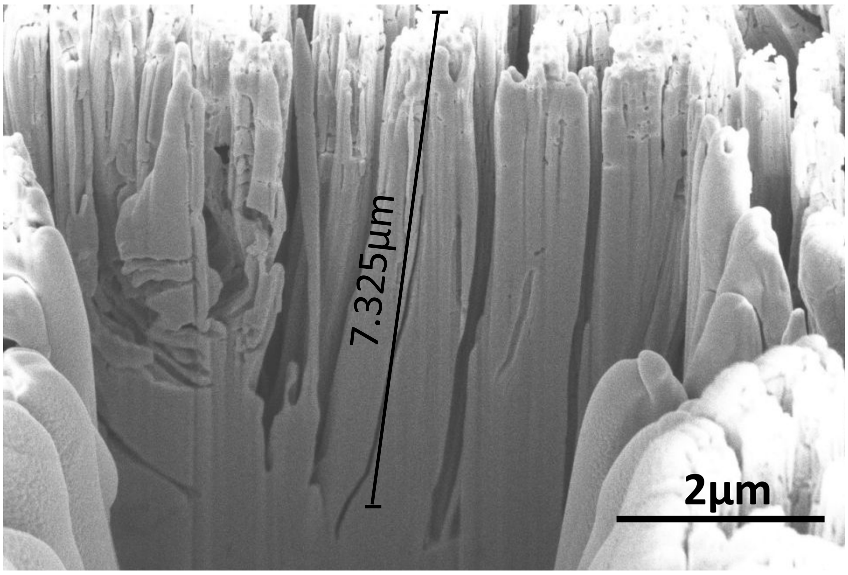

Figure S1 | FIB cross-section image of the SiNP using ion spattering, the resulting SiNP has a height of approximately 7µm.

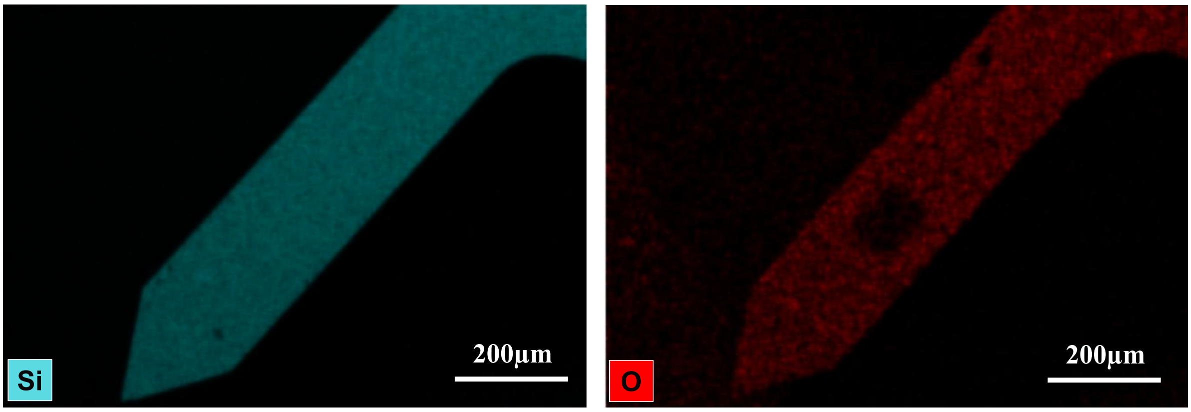

Figure S2 | EDS characterization of the silica protection layer of nanopillars sensing area. **(a)** Silicon is present on the entire needle area. **(b)** Oxygen presence was excluded from the sensing area.

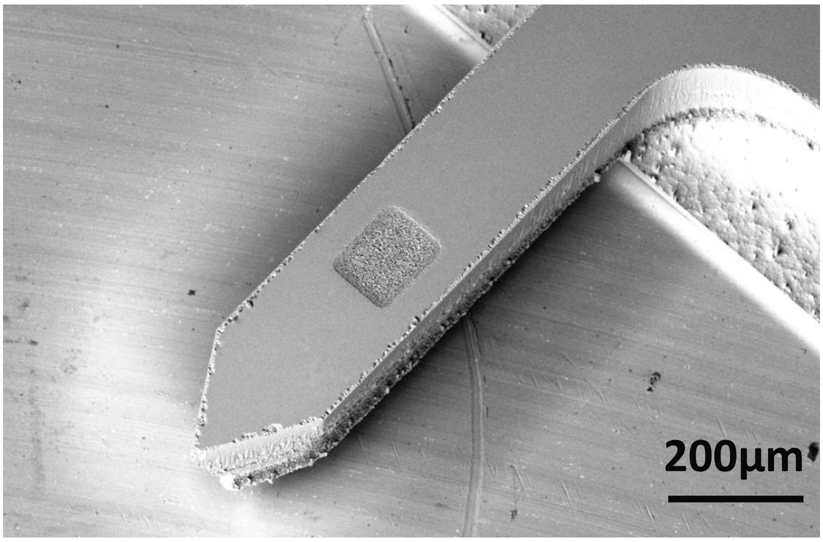

Figure S3 | BSD image of the needle showing shadowing effect around the SiNP sensing area as a result of the added protective layer of silica.

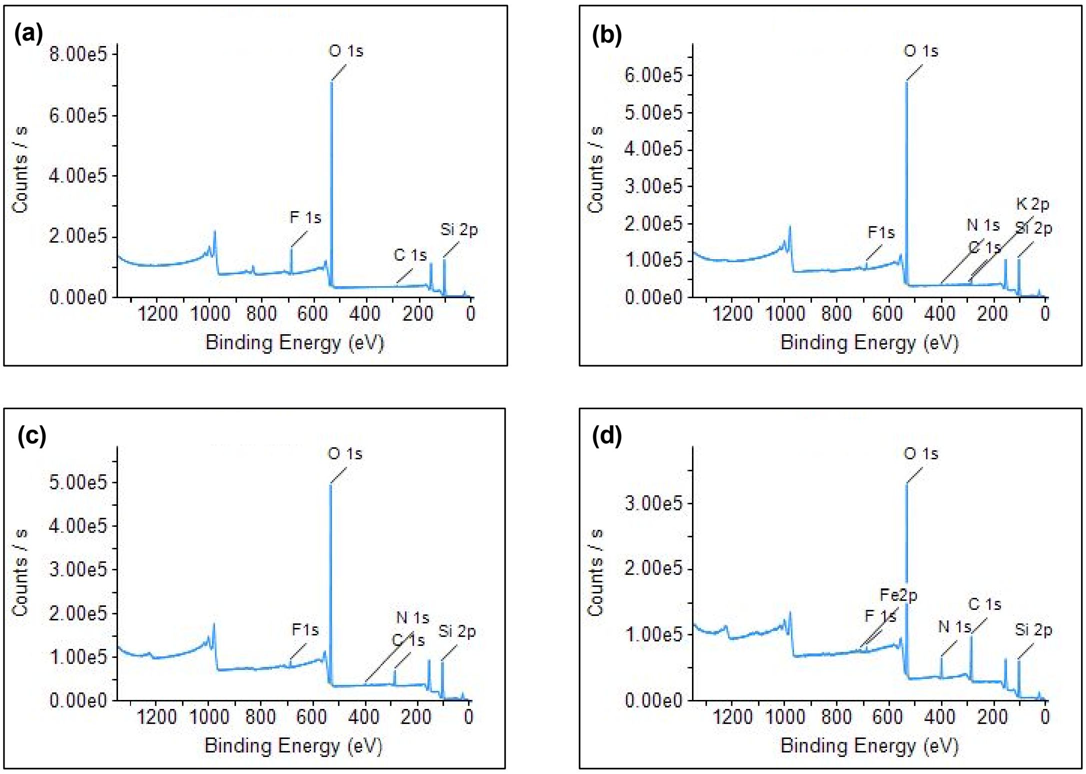

Figure S4 | Full XPS spectra of the antibody modification process to the SiNP.**(a)** Clean silicon wafer after oxygen plasma cleaning. **(b)** wafer modifies with APDMES for 2 h. **(c)** Wafer modified with glutaraldehyde, reduced to the surface with cyanoborohydride **(d)** wafer modified with IgG antibody.

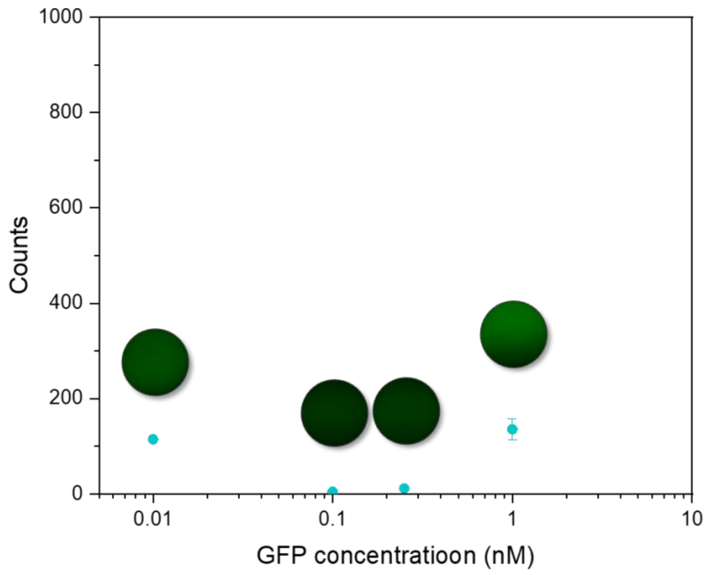

Figure S5 | Concentration dependent fluorescence measurement on a plain, GFP modified silicon wafer.

.

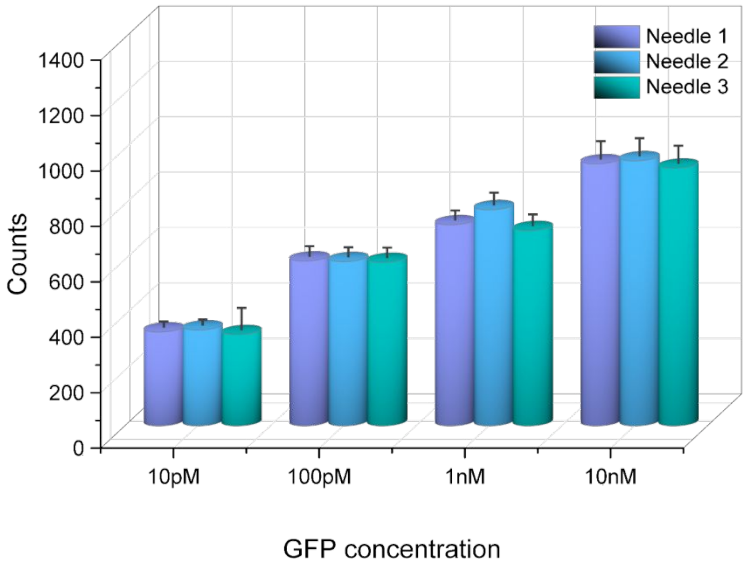

Figure S6 | Repeatability intensity measurements of the needles on a single chip under rising GFP concentrations. The results suggest that on a single chip, the measured intensity on all the needles is the same inside the error range.

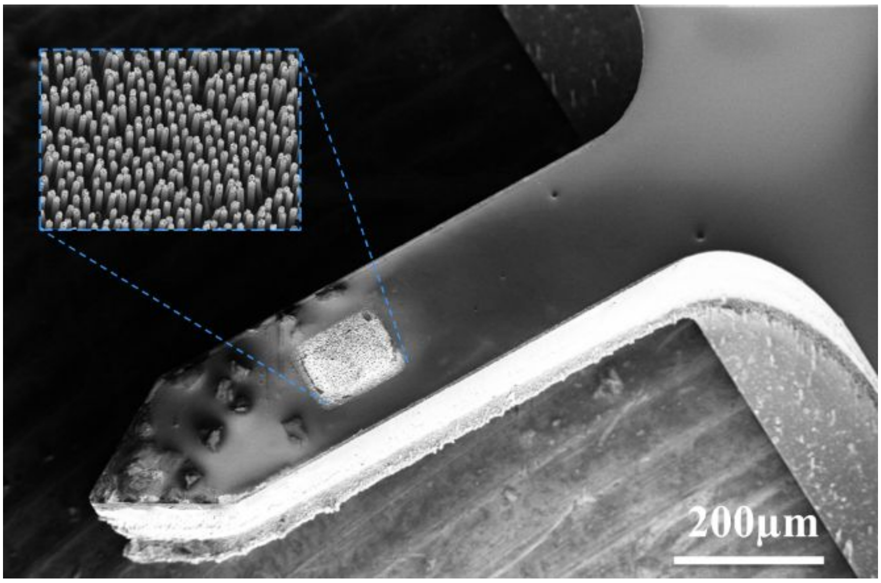

Figure S7 | SEM imaging of the needles and the pillars post skin pricking, minor skin residues were observed but no damage to the pillars was perceived.

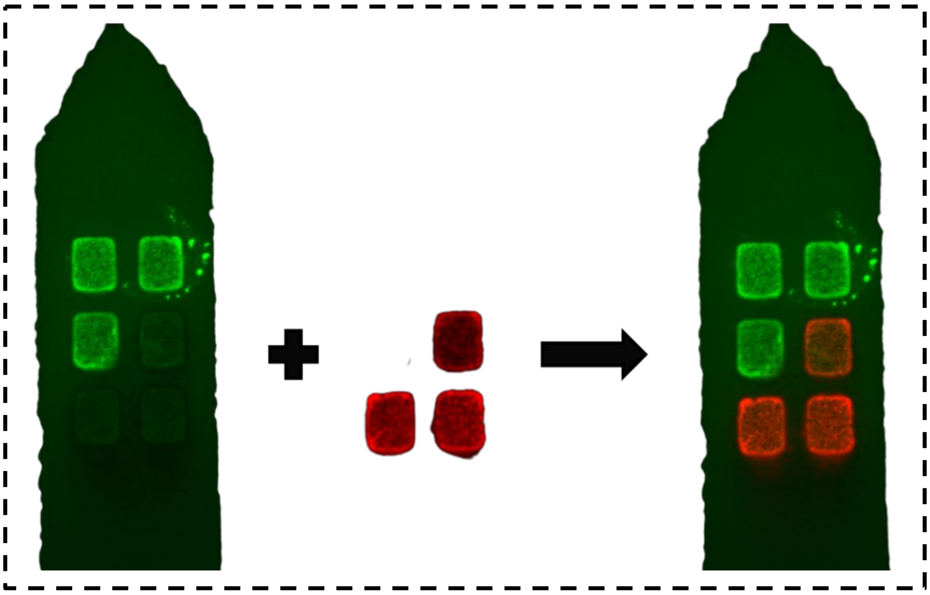

Figure S8 | The raw fluorescent images for multiplex detection. Both Alexa Fluor 430 and Alexa Fluor 555 were exited with the appropriate wavelength consequently resulting in a merged image of both channels.

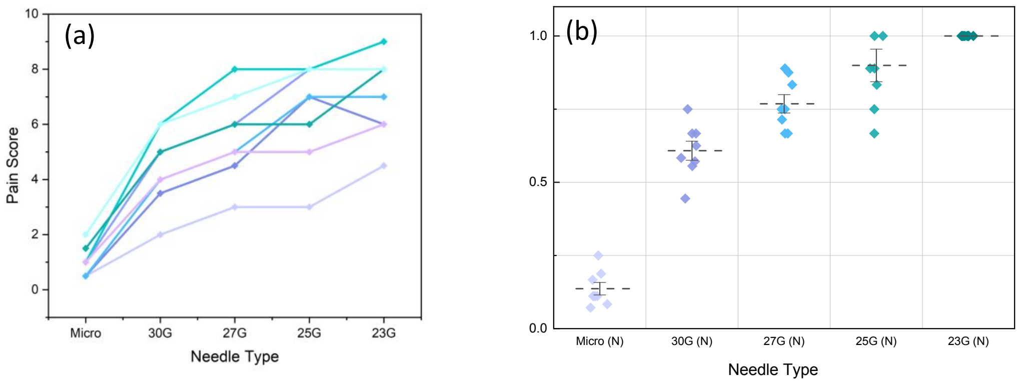

Figure S9 | Pain Test Survey presenting normalized pain reported from volunteers after finger pricking with the presented microneedle, 30G needle, 27G needle, 25 needle and 23G. (a) Raw data of pain scores from volunteers. (b) Normalized pain scores according to the 23G needle from each volunteer.

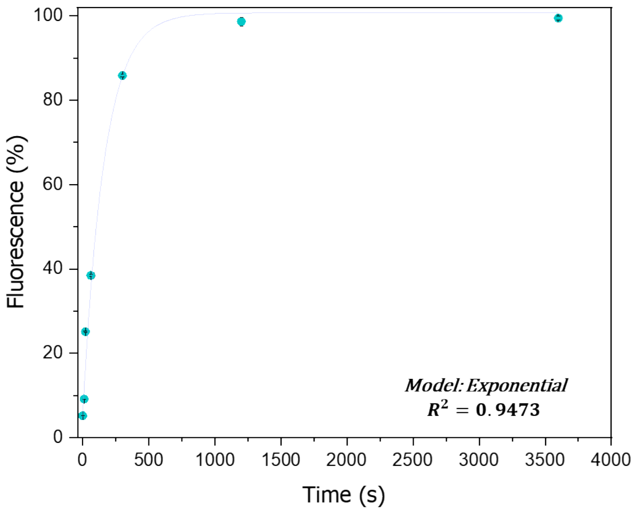

Figure S10 | Incubation time-dependence of binding of a secondary antibody to the protein on the pillars by fluoresce measurements.

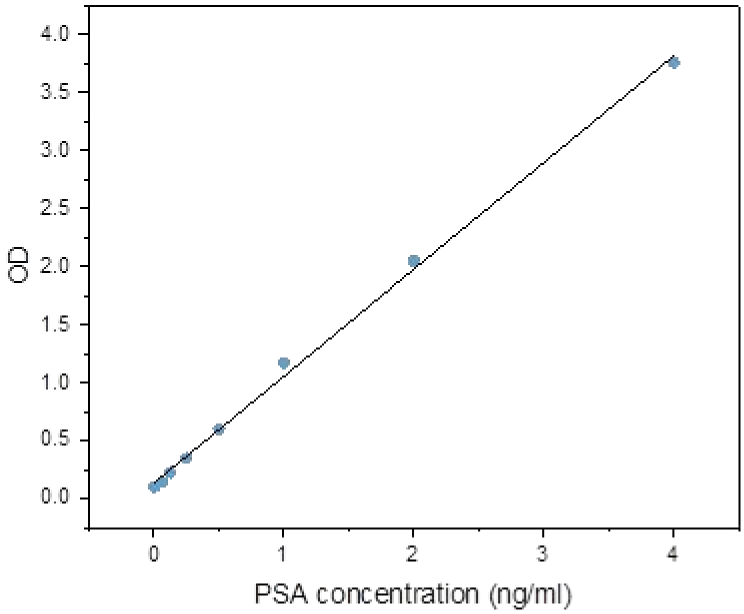

Figure S11 | Enzyme-linked immunosorbent assay calibration curve for PSA concentrations.

Table S1 | Fluorescence reproducibility from multiple chips with single GFP concentration (GFP concentration: 0.03nM).

Chip Number	1	2	3	4	5	6	7	8	9	10	Average/ Standard deviation
Counts	511.61	512.29	522.01	518.01	527.12	517.24	511.19	511.64	514.32	500.07	514.55/ 6.89
Average deviation	41.3	43.3	40.9	42.1	39.9	37.0	39.4	37.5	40.2	41.1	--

Supplementary Movie Legends

Movie S1 | The insertion process of the microneedle device in PDMS demonstrating all three needles on the device are fully intact following this process.

Movie S2 | An optical microscope video of a blood droplet introducing to a needle, the blood droplet draining into the depressed SiNPs array, fully saturating the sensing area.
