# Supplementary material for: Transdermal Minimally Invasive Optical Multiplex Detection of Protein Biomarkers by Nanopillars Array-Embedded Microneedles
Source: ACS Nano. 2024 Oct 28;18(44):30848–62. doi: 10.1021/acsnano.4c11612 (PMC11544710; doi:10.1021/acsnano.4c11612)
Supplement: Supplementary file 4 — nn4c11612_si_004.pdf [file nn4c11612_si_004.pdf]

CONFIDENTIAL

EVALUATION OF CYTOTOXIC EFFCT OF A TEST ITEM ON L929 FIBROBLASTS IN VITRO

Study No: FEP-001-IVT

Proposal No: SAF6147A

1. TEST SYSTEM

1.1 **Cells**: L929 (mouse fibroblasts, subcutaneous connective tissue; adipose; areolar)

1.2 **Test Items:** Chip

1.3 **Negative control:** untreated cells

1.4 **Assay**: Cell Proliferation Kit (XTT based) (Biological industries, Catalogue #: 20-300-1000)

2. EXPERIMENTAL DESIGN

2.1 L929 cells were plated on 96 well plate, in their culture medium, at $1x10^4$cells/well.

2.2 Cells were allowed to attach for 18 ±2 hours at 37ºC, 5% CO_2.

2.3 Thereafter, the culture medium was discarded, and 200 µL of culture medium + Test Item (6 wells), or culture medium alone as control (6 wells), were added to the cells.

2.4 The cells were incubated for 24 ±2 hours at 37ºC, 5% CO_2.

2.5 After incubation, qualitative evaluation of the cells was performed by microscopic grading according to the following score:

0- Noncytotoxic

1- Mildly cytotoxic

2- Moderately cytotoxic

3- Severely cytotoxic

2.6 Next, media was discarded, and 100 µL fresh culture medium was added to the cells along with 50 µL of XTT reagent.

2.7 The OD was measured using a plate reader, once untreated treated cells reached the range of 0.5-1.5 OD at 450 nm wavelength (after subtraction of the non-specific OD at 620 nm).

3. MATERIALS AND FORMULATIONS

3.1 L929 culture medium:

EMEM medium (Biological Industries, Cat# 01-025-1A) supplemented with 10% FBS (Biological Industries, Cat# 04-127-1A), 4mM L-Glutamine (Biological Industries, Cat# 03-020-1B), 1% Penicillin-Streptomycin solution (Biological Industries, Cat# 03-031-1B).

3.2 Cell Proliferation Kit (XTT based):

XTT reagent solution and the activation solution were defrosted immediately prior to use at 37°C. The solutions were swirled gently until a clear solution is obtained. To prepare a reaction solution sufficient for one plate (96 wells), 0.1 mL activation solution was added to 5 mL XTT reagent.

4. RESULTS

As seen in Figure 1, no significant differences were observed in the viability of untreated cells and cells incubated with the Test Items. In addition, microscopic evaluation did not point to a difference between untreated and Test Items treated cells, and all wells received 0 in the microscopic grading scale.

Raw data can be found in Table 1.

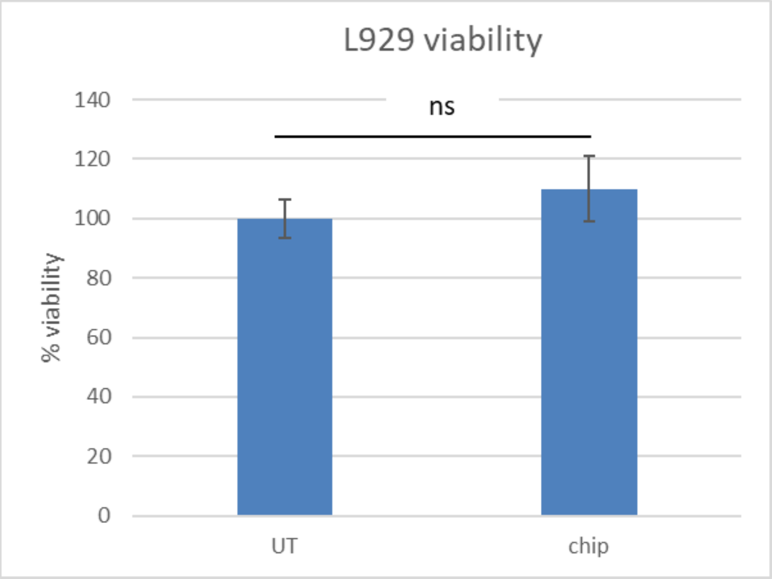

Figure 1: Effect of Test Items on L929 cells viability. L929 cells were treated with Test Items. Untreated cells served as control. Cell viability was determined by XTT assay. Results represent means ±SEM of wells in each group.

Table 1: L929 cells viability raw data

	1	2	3	4	5	6	7	8	9	10	11	12	
A	0.005	0.015	0.006	0.004	0.009	0.004	0.004	0.006	0.004	0.006	0.006	0.009	Delta
B	0.14	0.682	0.72	0.75	0.068	0.069	0.069	0.068	0.006	0.008	0.007	0.009	Delta
C	0.141	0.652	0.669	0.742	0.071	0.071	0.07	0.072	0.008	0.005	0.005	0.007	Delta
D	0.141	0.648	0.714	0.751	0.069	0.071	0.072	0.07	0.005	0.005	0.005	0.004	Delta
E	0.14	0.68	0.738	0.758	0.071	0.075	0.071	0.068	0.003	0.005	0.004	0.004	Delta
F	0.144	0.707	0.848	0.779	0.073	0.071	0.07	0.073	0.004	0.005	0.004	0.005	Delta
G	0.138	0.743	0.748	0.777	0.073	0.069	0.068	0.071	0.005	0.006	0.005	0.007	Delta
H	0.007	0.005	0.005	0.004	0.008	0.003	0.004	0.006	0.005	0.006	0.005	0.008	Delta

		OD			% Viability	
background		UT	chip		UT	chip
0.140667		0.541	0.579		99.4	106.4
		0.511	0.528		93.9	97.0
		0.507	0.573		93.1	105.3
		0.539	0.597		99.0	109.7
		0.566	0.707		104.0	129.9
		0.602	0.607		110.6	111.5
				avg	100.0	109.9
				sd	6.5	11.0
					t-test	0.086

Draft Histopathology Report

Study Number: PHS-1791a+b-HIS

Pharmaseed Study No.: FEP-002-SAF

All rights reserved.

No part of this publication may be reproduced and/or published by print, photoprint, microfilm, or any other means, without the previous written consent of PATHO-LOGICA.

© 2016 PATHO-LOGICA

Patho-Logica

PATHO-LOGICA Ltd	PATHOLOGY REPORT
Study Report Status	Draft Histopathological Report
Study Number	PHS-1791a+b-HIS
Pharmaseed Study Number	FEP-002-SAF
Study Schedule	Study Starting date: 07/04/2024<br>Draft Report date: 14/04/2024<br>Final Report date:
**Study Title**	**Organ's evaluation in mouse: histological assessment**
Test site	Patho-Logica,<br>3 Golda Meir, St.<br>Scientific Park, Ness Ziona<br>7403648, Israel<br>WWW.patho-logica.com
Author	Dr. Emmanuel Loeb, Veterinary Pathologist
Prepared for	Perri Rozenberg-Hasson, PhD<br>P: +972 8 9302771 ext. 201<br>M: +972 54 4493514<br>perri@pharmaseedltd.com<br>Website: http://www.pharmaseedltd.com/<br>Twitter: https://twitter.com/pharmaseedltd<br>Linkedin: https://www.linkedin.com/company/pharmaseed-ltd

Patho-Logica

Table of contents

ABBREVIATIONS **4**

DECLARATIONS **5**

MATERIALS AND METHODS **6**

a. Study Design 6

b. Organ/Tissue Collection and Fixation 6

c. Slide Preparation 6

d. Light Microscopy Photography 6

e. Histological Evaluation 6

RESULTS **6**

Histology 7

Representative Histologic Photographs 7

SUMMARY AND CONCLUSIONS **11**

ARCHIVING **11**

REFERENCES **11**

Abbreviations

GLP	Good Laboratory Practice
H&E	Hematoxylin & Eosin
OECD	Organization for Economic Co-Operation and Development
SD	Standard deviation

Declarations

Histopathology Evaluation Report - Declaration and Signature

I hereby declare that the histological evaluation of this study was performed and reported based on the OECD principles of Good Laboratory Practice ENV/MC/CHEM (98)17. However, it does not fully comply with GLP regulations and thus is considered a non-GLP study.

The tested items are original materials provided by the Sponsor and analysed by Patho-Logica Ltd.

The evaluation was conducted by a veterinary pathologist expert, licence no. 436.

____________________ ____________________

Emmanuel Loeb **Date**

Vet. Path Specialist.

Patho-Logica

Objective

The objective of the present study was to evaluate safety histological parameters in untreated animal organs using a semi-quantitative analysis.

Materials and Methods

a. Study Design

Table 1. Study design

Animal No.	Group No,	No. of Animals	Treatment	Sacrifice Date
1, 5	1F	2	untreated	
2, 3, 4	2F	3	2, 3 (unmodified)<br>4 (modified)	
**Total**		5		

b. Organ/Tissue Collection and Fixation

Samples of Heart, Lungs, Kidneys, Liver, Brain, Spleen and Skin tissue from five mice were harvested, fixed in 4% formaldehyde, arrived to Patho-Logica, and left in fixative for 48 hours. The tissues were trimmed, put in embedding cassettes, and processed routinely for paraffin embedding. Five cassettes were prepared per animal.

c. Slide Preparation

Paraffin sections, 4 microns thick were cut, put on glass slides, and stained with Hematoxylin & Eosin (H&E).
The slides were subjected to histological evaluation by Dr. Loeb.
Stained slides will be available for the Sponsor upon request.

d. Light Microscopy Photography

Pictures were taken using Olympus microscope (BX60, serial NO. 7D04032) equipped with microscope's Camera (Olympus DP73, serial NO. OH05504) at objective magnifications of XXX.

e. Histological evaluation

The stained slides were examined and scored by the study Pathologist, using a semi-quantitative scoring scale of five grades (0-4), for the severity of the pathological changes (Schafer et al.):
Grade 0 – Normal.
Grade 1 – Minimal pathological findings.
Grade 2 – Mild pathological findings.
Grade 3 – Moderate pathological findings.
Grade 4 – Severe pathological findings.

Results

Histopathology

Histopathology

- In general, five animals, three tested and two untreated, we evaluated for possible pathological changes on seven different tissues (Heart, lungs, kidneys, liver, brain, spleen, and skin) using histological tools.
- In all tested animals we observed no histopathological changes, and all evaluated tissues had a normal appearance.
- Pathological changes are described below in Table B:

Table 2. A summary of the semi-quantitative analysis of the histological findings, using a scoring scale (see details in M&M)

Group/ treatment	Animal No.	Heart	Lungs	Kidneys	Liver	Brain	Spleen	Skin	Comments
**1F**	1	0	0	0	0	0	0	0	
	5	0	0	0	0	0	0	0	
N=2		**0**	**0**	**0**	**0**	**0**	**0**	**0**	
**2F**	2 (unmodified)	0	0	0	0	0	0	0	
	3 (unmodified)	0	0	0	0	0	0	0	
	4 (modified)	0	0	0	0	0	0	0	
N=3		0	0	0	0	0	0	0	

Representative Histological Photographs

H&E stained slides; Organs –X10

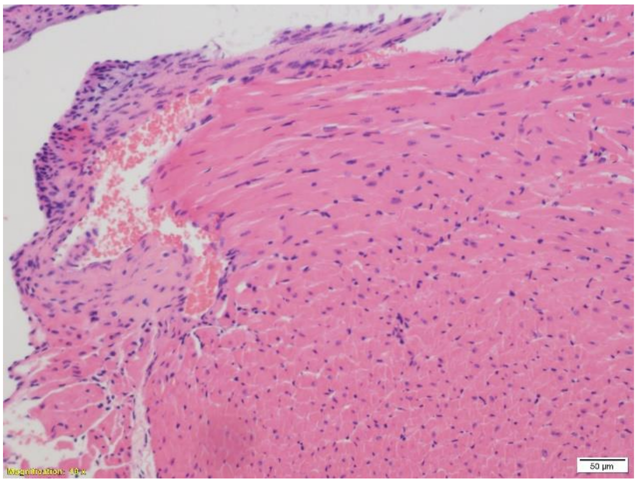

Figure 1. Heart. Group 1F. Animal 1. Junction between the ventricle and atrium. Tissue unaffected. X10. H&E.

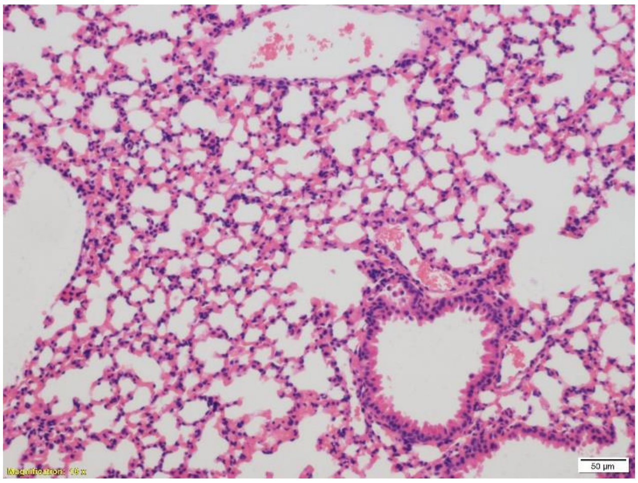

Figure 2. Lung. Group 1F. Animal 1. Tissue unaffected. X10. H&E.

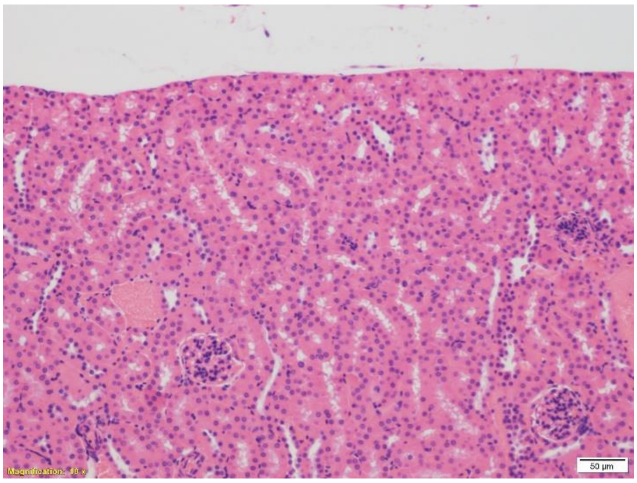

Figure 3. Kidney. Group 1F. Animal 1. Tissue unaffected. X10. H&E.

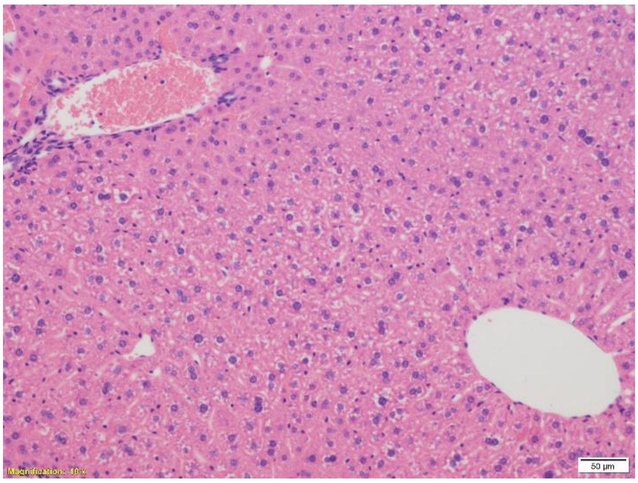

Figure 4. Liver. Group 1F. Animal 1. Tissue unaffected. X10. H&E.

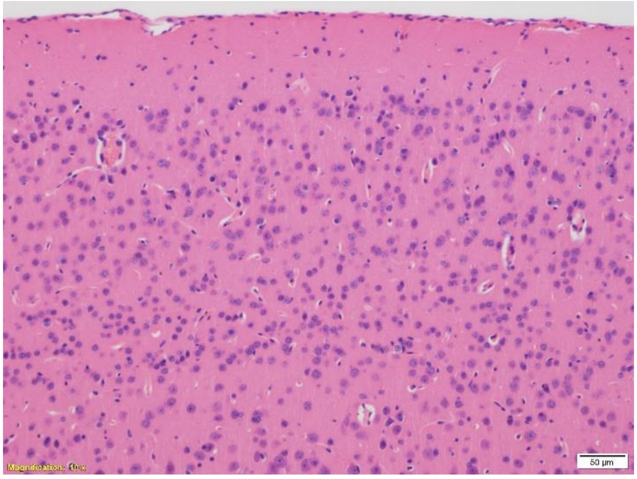

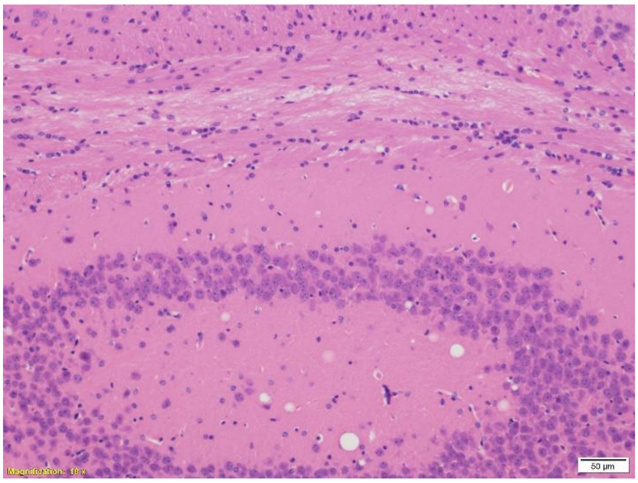

Figure 5. Brain (cortex). Group 1F. Animal 1. Tissue unaffected. X10. H&E.

Figure 6. Brain (hippocampus). Group 1F. Animal 1. Tissue unaffected. X10. H&E.

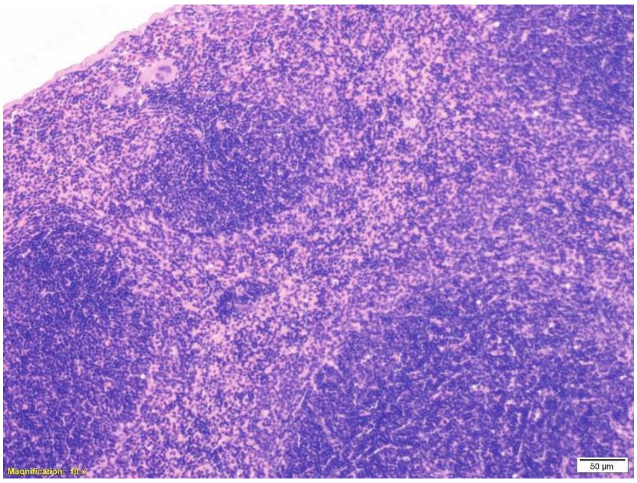

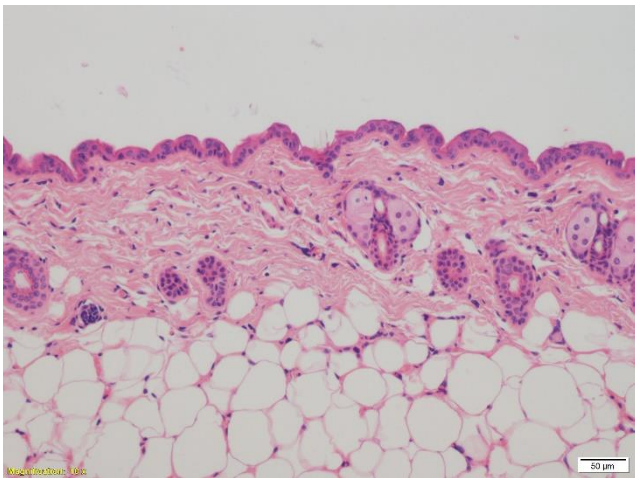

Figure 7. Spleen. Group 1F. Animal 1. Tissue unaffected. X10. H&E.

Figure 8. Skin. Group 1F. Animal 1. Tissue unaffected. X10. H&E.

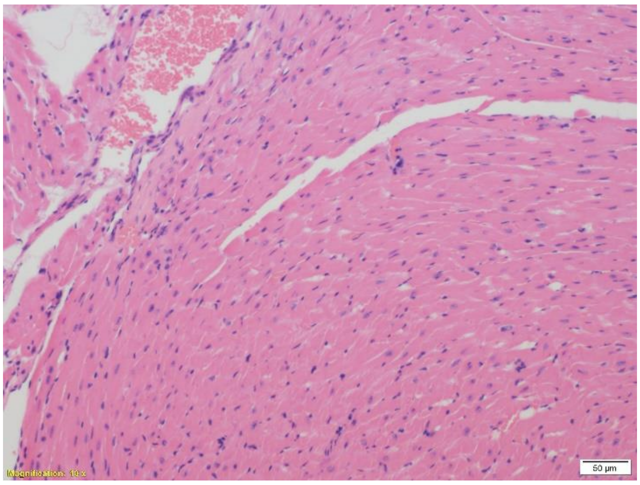

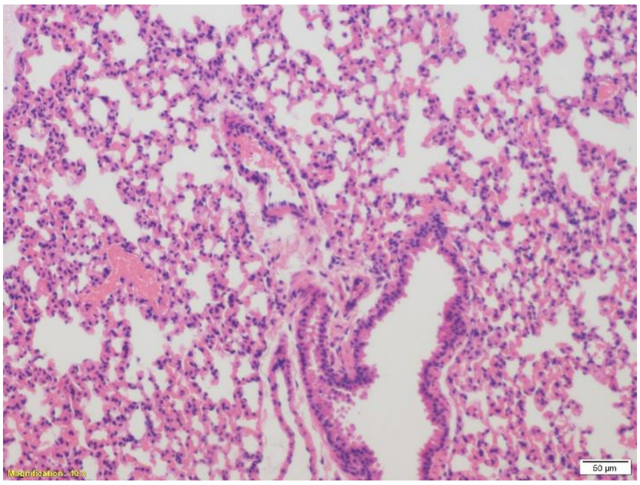

Figure 9. Heart. Group 2F. Animal 4. Junction between the ventricle and atrium. Tissue unaffected. X10. H&E.

Figure 10. Lung. Group 2F. Animal 4. Tissue unaffected. X10. H&E.

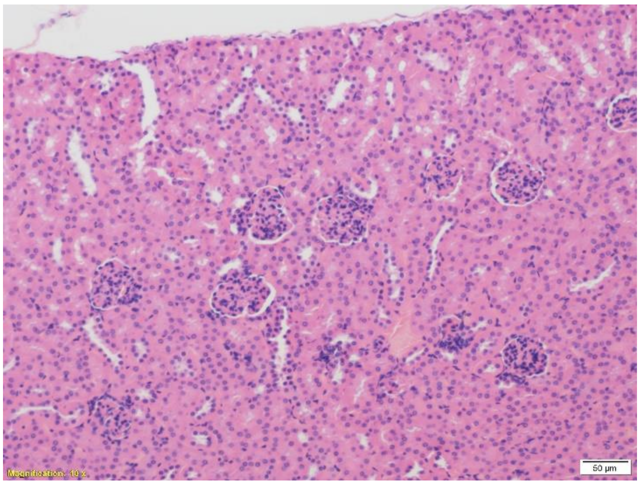

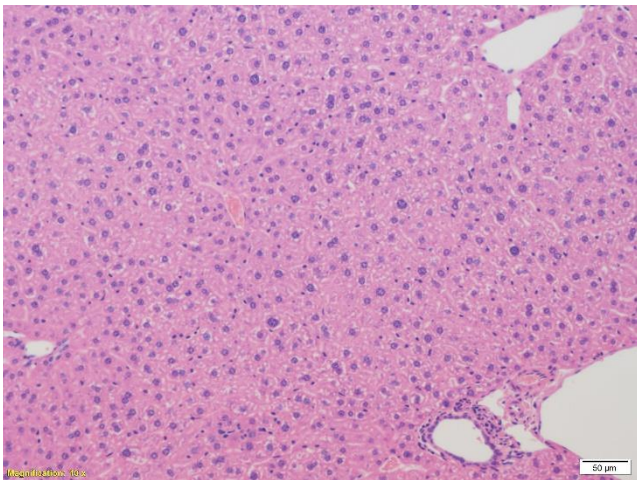

Patho-Logica

Figure 11. Kidney. Group 2F. Animal 4. Tissue unaffected. X10. H&E.

Figure 12. Liver. Group 2F. Animal 4. Tissue unaffected. X10. H&E.

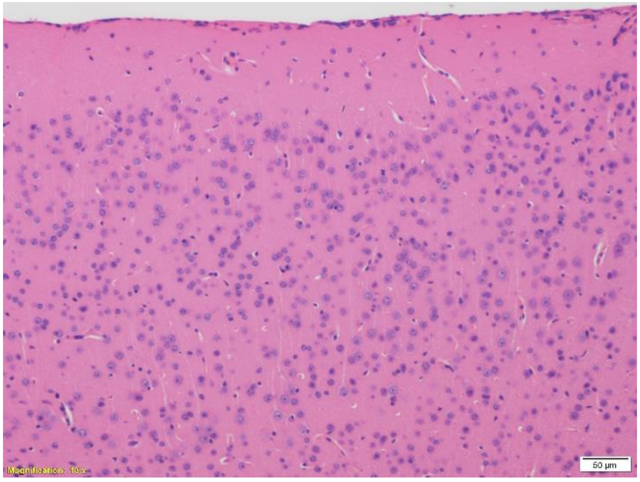

Figure 13. Brain (cortex). Group 2F. Animal 4. Tissue unaffected. X10. H&E.

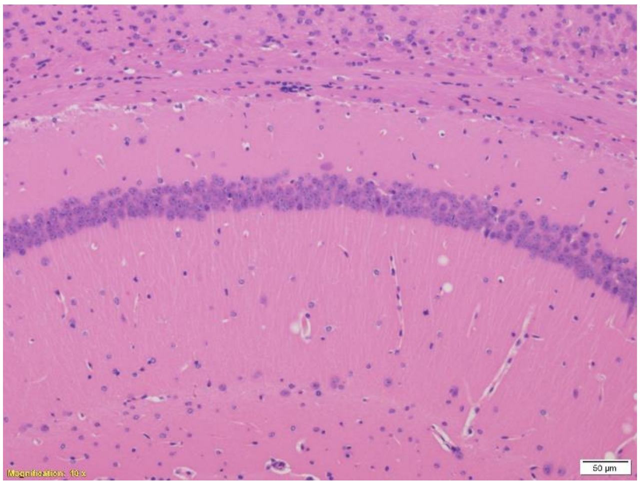

Figure 14. Brain (hippocampus). Group 2F. Animal 4. Tissue unaffected. X10. H&E.

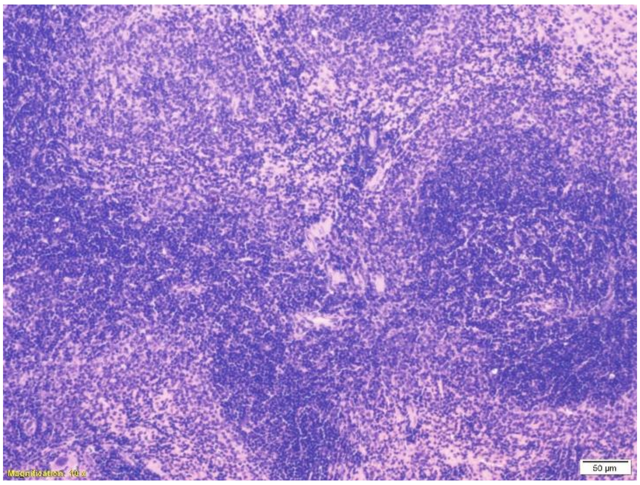

Figure 15. Spleen. Group 2F. Animal 4. Tissue unaffected. X10. H&E.

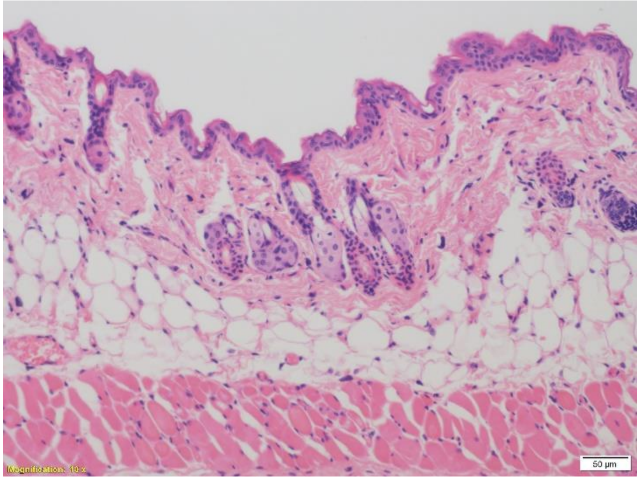

Figure 16. Skin. Group 2F. Animal 4. Tissue unaffected. X10. H&E.

Summary and Conclusions

Sections of five animals organs (heart, lungs, kidneys, liver, brain, spleen, and skin) were stained with H&E and evaluated for any histopathological findings. We observed no apparent lesions or any histopathological changes in this animal.

Archiving

The final Study Plan, final Study Report, study related records, test item data, and relevant correspondence will be retained in the document archive for a period of one year.

All the study slides will be retained in the slide archive for a period of one year.

All the study paraffin blocks, will be retained in the block archive for a period of one year.

During this period a request for unstained slides may be submitted and funded. At the end of that time period the Sponsor will be contacted and requested to receive the archived material, agree in writing to its disposal or leave it at Patho-Logica Archive for an annual fee.

REFERENCES

1. Kerlin R, Bolon B, Brukhardt J, Francke S, Greaves P, Meador V, Popp J.: Scientific and Regulatory Policy Committee: Recommended ("Best") Practices for Determining, Communicating, and Using Adverse Effect Data from Nonclinical Studies. Toxicol Pathol. 2016 Feb; 44(2): 147- 62.
2. Schafer KA, Eighmy J, Fikes JD, Halpern WG, Hukkanen RR, Long GG, Meseck EK, Patrick DJ, Thibodeau MS, Wood CE, Francke S. (2018) Use of Severity Grades to characterize Histopathologic Changes. Toxicol Pathol.; 46(3): 256-265.
